# Microscopic versus Macroscopic Glass Transitions and Relevant Length Scales in Mixtures of Industrial Interest

**DOI:** 10.1021/acs.macromol.2c02368

**Published:** 2023-02-28

**Authors:** Numera Shafqat, Angel Alegría, Nicolas Malicki, Séverin Dronet, Francesca Natali, Lucile Mangin-Thro, Lionel Porcar, Arantxa Arbe, Juan Colmenero

**Affiliations:** † Centro de Física de Materiales (CSIC, UPV/EHU) and Materials Physics Center MPC, Paseo Manuel de Lardizabal 5, E-20018 San Sebastián, Spain; ‡ Manufacture Française des Pneumatiques MICHELIN, Site de Ladoux, 23 place des Carmes Déchaux, F-63040 Cedex 9, Clermont-Ferrand, France; § Departamento de Polímeros y Materiales Avanzados: Física, Química y Tecnología (UPV/EHU), Apartado 1072, E-20018 San Sebastián, Spain; ∥ CNR-IOM, OGG, 71 avenue des Martyrs, 38043 Cedex 9, Grenoble, France; ⊥ Institut Laue-Langevin, 71 avenue des Martyrs, 38042 Cedex 9, Grenoble, France; # Donostia International Physics Center (DIPC), Paseo Manuel de Lardizabal 4, E-20018 San Sebastián, Spain

## Abstract

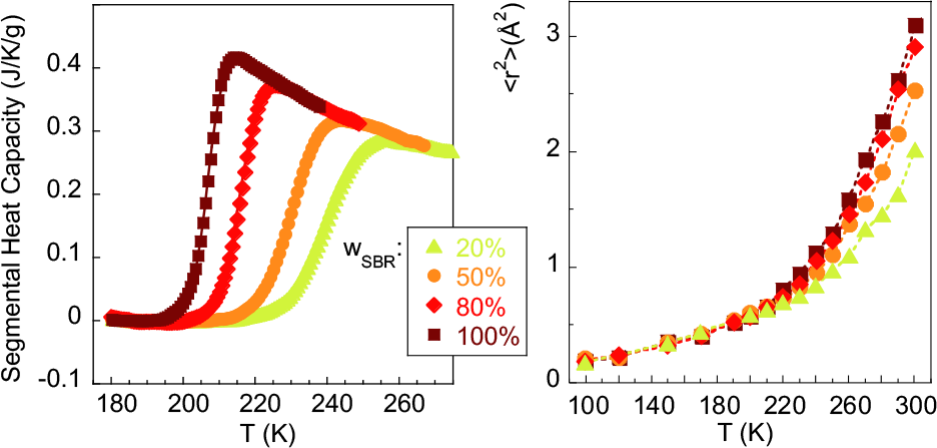

We have combined X-ray diffraction, neutron diffraction with polarization analysis, small-angle neutron scattering (SANS), neutron elastic fixed window scans (EFWS), and differential scanning calorimetry (DSC) to investigate polymeric blends of industrial interest composed by isotopically labeled styrene–butadiene rubber (SBR) and polystyrene (PS) oligomers of size smaller than the Kuhn length. The EFWS are sensitive to the onset of liquid-like motions across the calorimetric glass transition, allowing the selective determination of the “microscopic” effective glass transitions of the components. These are compared with the “macroscopic” counterparts disentangled by the analysis of the DSC results in terms of a model based on the effects of thermally driven concentration fluctuations and self-concentration. At the microscopic level, the mixtures are dynamically heterogeneous for blends with intermediate concentrations or rich in PS, while the sample with highest content of the fast SBR component looks as dynamically homogeneous. Moreover, the combination of SANS and DSC has allowed determining the relevant length scale for the α-relaxation through its loss of equilibrium to be ≈30 Å. This is compared with the different characteristic length scales that can be identified in these complex mixtures from structural, thermodynamical, and dynamical points of view because of the combined approach followed. We also discuss the sources of the non-Gaussian effects observed for the atomic displacements and the applicability of a Lindemann-like criterion in these materials.

## Introduction

The glass transition phenomenon arises as a loss of equilibrium of the so-called α-relaxation in a supercooled liquid upon cooling. It manifests as a step in the specific heat measured by differential scanning calorimetry (DSC). At the glass-transition temperature *T*_g_ observed by DSC, the characteristic time of the α-process as monitored by relaxation techniques like broad band dielectric spectroscopy (BDS) assumes values of the order of seconds. Despite the great effort performed by the scientific community over the past decades, the vitrification phenomenon is not yet completely understood. Nevertheless, materials with enhanced properties are constantly designed driven by industrial demand. Particularly interesting are mixtures of polymers where the *T*_g_s of the neat components are very different—so-called dynamically asymmetric mixtures—offering thereby a great tunability of the properties of the resulting compound by playing with the composition. These polymer blends exhibit a complex phenomenology.^1,2^ One main experimental observation in polymer blends is the dynamic heterogeneity (finding of two different characteristic times associated with two distinct α-relaxations of the two components). This property—which implies the presence of two different *T*_g_s in the blend—is thought to be a consequence of self-concentration (SC) effects, i.e., to the fact that the local concentration around one segment of one of the blend components is always richer in this component due to chain connectivity. The other main experimental finding is the broadening of the measured relaxation functions, which is attributed to thermally driven concentration fluctuations (TCF). These two ingredients, SC and TCF, were incorporated in a model developed by us to describe BDS and mechanical results^3,4^ on mixtures. The systems investigated in those works were blends of styrene–butadiene rubber (SBR) and polystyrene (PS). SBR and PS display very different values of their *T*_g_s, that of PS being much higher. Miscibility of these two polymers is only possible if PS oligomers are considered. Mixtures of SBR and oligomers of PS can be viewed as simplified systems in the tire industry because they simultaneously fulfill the desired decrease in rolling resistance and increase of energy dissipation during braking to optimize tire performance. Using PS oligomers, the high-*T*_g_ component acts as a “plasticizer”. In practice, it is observed that when using these oligomers, the grip performance is improved. It is also important to emphasize that even if the PS is not employed for tire materials, its *T*_g_ and molar mass are very similar to the ones of real plasticizers used in the industry, and for that purpose, PS is very suitable as a model system. Recently, we could also predict the calorimetric response extending the same model to DSC results on these mixtures and taking as input the information obtained by BDS on the α-relaxations behavior in equilibrium and by small-angle neutron scattering (SANS) on the TCF.^5^ Noteworthy, the model allows to extract the two contributions to the calorimetric trace, i.e., the “effective *T*_g_” of each of the blend components (*T*_g,eff_^SBR^ and *T*_g,eff_^PS^ in the case of those blends).

In addition to macroscopic methods like BDS, mechanical spectroscopy, or DSC, scattering techniques provide a great help to unveil the properties of the system at the microscopic level. These techniques access spatial information at this level through the analysis of the scattering vector (*Q*) dependence of the scattered intensity. Several kinds of scattering experiments can shed light on different aspects of the behavior of complex systems like polymer blends:

(i) From a structural point of view, diffraction experiments at high *Q* (around 1 Å^–1^), i.e., exploring local length scales of the order of the typical intermolecular distances), inform about the short-range order of the material. This information is provided by neutron as well as X-ray diffraction, with different weights of the pair correlation contributions to the structure factor. In the case of the PS oligomers previously investigated (molecular weight of 900 g/mol), as well as in the corresponding blends with SBR, this kind of experiment revealed the presence, together with the usual main peak at about 1.3 Å^–1^ related to intermolecular correlations, of a “prepeak”. This feature was tentatively attributed to the existence of nanodomains arising from the nanosegregation of main-chain and phenyl ring atoms already observed in high-molecular-weight PS.^6^
(ii) Small-angle scattering experiments exploring the low-*Q* region (equivalently, large length scales) offer the direct observation of TCF in mixtures, revealing the amplitude and correlation length of these fluctuations. These magnitudes reflect the *thermodynamics* of the system, allowing to determine the phase diagram; but also, as mentioned above, TCF are believed to be responsible for the broad *dynamic* response of the blend components as reflected by the macroscopic techniques addressing relaxational processes. These kinds of studies are performed using neutrons as probe (SANS) and enhancing the contrast between the two components by mixing protonated and deuterated chains.
(iii) Also employing neutrons and through the analysis of their energy transfer with the sample, quasi-elastic neutron scattering experiments allow obtaining dynamic information. In the case of blends, there is the possibility of “labeling” a given component and selectively following its microscopic dynamics at local length scales—in particular, the self-motions of its hydrogens—by deuterating the other component. The time range explored in this kind of experiments is of the order of picoseconds to nanoseconds.

In connection with the question mentioned above about the unsolved problem of the glass transition in glass-forming systems in general, we note that the microscopic information provided by neutron scattering on the atomic motions can be of utmost help. In this direction, we recall a recurrent observation when the atomic displacements at times of the order of tens of picoseconds to nanoseconds are monitored in “simple” (no mixtures) glass-forming systems: a clear change of the behavior of this magnitude when crossing the macroscopic glass transition detected by DSC. This finding has been reported for systems of very different character, as e.g. in molecular liquids as in the initial work of Fujara and Petry^7^ and later by Alba-Simionesco et al.,^8^ or in polymers by Frick et al.,^9,10^ and can be considered as a support for theoretical frameworks for the glass transition as the so-called elastic models.^11,12^ The question arising when considering now a more complex system as a dynamically asymmetric mixture is: do the atomic displacements of a given component at short times still “feel” its macroscopically observed effective glass transition temperature?

In addition, the comparison of macroscopic and microscopic results on polymer blends can address the question of the relevant length scale involved in the glass transition. This is achieved through the observed broadening of the macroscopic signal due to TCF and because of the spatial information provided by SANS on the same phenomenon. In previous works this strategy has revealed this scale to be 1–2.5 nm for two families of blends containing the same deuterated PS with a molecular weight of 900 g/mol and SBR of different microstructures and molecular weights.^5,13^ Whether this range is also found for different systems is an open question.

With these ideas in mind, in this work we have performed a thorough investigation on similar blends, now composed by SBR with a different microstructure and an even smaller oligomer of PS of about 500 g/mol. With these samples we can check whether the model used in previous works also applies in mixtures with different characteristics in terms of SBR microstructure, PS size, and dynamic asymmetry between the components. We note that the smaller PS component improves miscibility, while still keeping a noticeable dynamic asymmetry in the system. We have applied different neutron scattering techniques: diffraction with polarization analysis, SANS, and elastic fixed window scans (EFWS), the latter addressing atomic motions at the microscopic level and thereby heterogeneities and non-Gaussian effects. Using X-ray diffraction, we have checked also in these samples the presence of nanodomains. In addition, we have performed in parallel a full DSC analysis in terms of the above-mentioned model. X-ray diffraction results and DSC analysis are presented in the Supporting Information for the sake of space in the article. With this combined approach, we have been able to determine and compare the different length scales that are relevant from structural, thermodynamical, and dynamical points of view in these complex mixtures.

## Experimental Section

### Samples

Protonated and deuterated styrene–butadiene rubber (hSBR and dSBR, respectively) were synthesized by anionic polymerization by the Michelin Company.^14^ The copolymerization was initiated by BuLi in methylcyclohexane at 50 °C. The polystyrene samples (hPS and dPS for the protonated and deuterated samples, respectively) were purchased from Polymer Source. They were synthesized by living anionic polymerization. Table 1 shows the molecular weights and microstructural composition of the samples. Though the molecular weights of the SBR samples are different, both are high enough to discard possible impact of the size of these macromolecules on their segmental dynamics. Parameters of interest for the SANS investigation are compiled in Table 2. As shown by infrared (IR) spectroscopy, while the deuteration level of dSBR was higher than 95%, the end groups of dPS were hydrogenated; magnitudes of relevance for the scattering experiments have been calculated accordingly.

**Table 1:** Molecular Weights, Polydispersities, and Densities of the Neat Components Investigated, and Weight Percentages of Styrene (S), 1,2-Butadiene (1,2-B), and 1,4-Butadiene (1,4-B) Monomers in the SBR Samples

sample	*M*_n_ (g/mol)	*M*_w_ (g/mol)	PDI	*d* (g/cm^3^)	wt % S	wt % 1,2-B	wt % 1,4-B
hSBR	69900	76200	1.09	0.94	13.8	21.8	64.4
dSBR	38100	43100	1.13	1.06	18.0	18.9	63.1
hPS	500	600	1.20	0.99			
dPS	500	550	1.12	1.07			

**Table 2:** Composition, Mass, Volume, Their Average Number in the Chains, Scattering Length *b* of the Effective Monomers, and Scattering Length Densities ρ for the Homopolymers

sample	effective monomer	*M*_o_ (g/mol)	*v* (cm^3^)	*N̅*	*b* (cm)	ρ (cm^–2^)
hSBR	[C_8_H_8_]_0.077_[C_4_H_6_]_0.923_	57.85	1.022 × 10^–22^	1317	0.5632 × 10^–12^	5.512 × 10^9^
dSBR	[C_8_D_8_]_0.105_[C_4_D_6_]_0.895_	65.46	1.025 × 10^–22^	658	7.081 × 10^–12^	69.07 × 10^9^
hPS	[C_8_H_8_]_0.81_[C_4_H_10_]_0.19_	95.26	1.580 × 10^–22^	6.3	1.680 × 10^–12^	10.61 × 10^9^
dPS	[C_8_D_8_]_0.80_[C_4_H_10_]_0.20_	101.20	1.570 × 10^–22^	5.4	8.309 × 10^–12^	52.91 × 10^9^

Blends of different compositions, where one of the components was protonated and the other deuterated, were prepared by solution casting using tetrahydrofuran (THF) as a solvent. The compositions were chosen such that the molar composition was the same independently of the isotopic label and corresponded to approximate SBR weight fractions of 80, 50, and 20% for the case of a mixture of fully protonated components. The obtained films were carefully dried under vacuum at 343 K for 24 h to remove the solvent completely. Reference samples of the neat polymers were prepared in a similar way. The nomenclature and composition of the samples investigated can be found in Table 3.

**Table 3:** Composition of the Samples Investigated and Ratio between Incoherent and Coherent Scattering Cross Sections

sample	wt % hSBR	wt % dPS	wt % dSBR	wt % hPS	σ_inc_/σ_coh_
hSBR	100	0	0	0	14.3
80h	77	23	0	0	10.8
50h	44	56	0	0	6.8
20h	19	81	0	0	3.9
dPS	0	100	0	0	2.2
dSBR	0	0	100	0	0.2
80d	0	0	82	18	1.8
50d	0	0	56	44	4.8
20d	0	0	22	78	8.8
hPS	0	0	0	100	12.2

### Differential Scanning Calorimetry (DSC)

DSC measurements were performed on samples of approximately 10 mg placed in aluminum pans using a Q2000 TA Instruments calorimeter. A liquid nitrogen cooling system (LNCS) was used with 25 mL/min helium flow rate. Data were acquired during cooling at 3 K/min from 353 to 93 K. Temperature-modulated experiments (MDSC) were performed using a sinusoidal variation of 0.5 K amplitude and 60 s period.

### Small-Angle Neutron Scattering (SANS)

SANS experiments on the blends listed in Table 3 were performed on the instrument D22 at the Institut Laue-Lagevin (ILL) in Grenoble, France.^15^ Using an incident wavelength λ = 6 Å and sample–detector distances (SSD) of 17, 5.6, and 1.5 m, a *Q* range between 0.003 and 0.58 Å^–1^ was covered. Here, the modulus of the scattering vector *Q* is defined as *Q* = 4πλ^–1^ sin(θ/2), with θ the scattering angle. The samples with thickness of 1 mm were sandwiched between aluminum foils. Experiments were performed first at 298 K. Then, the samples were heated at 385 K, i. e., well above the glass transition temperatures, and data were collected in isothermal conditions at 385, 327, 282, and 267 K. The data were reduced correcting measured intensities for the transmission, dead time, sample background, and detector background (with B4C as a neutron absorber at the sample position).

### Elastic Fixed Window Scans (EFWS)

In EFWS the energies of the incident and the detected neutrons after interacting with the sample are identical. The recorded intensity includes contributions with energy transfers smaller than the resolution of the spectrometer, δℏω. The EFWS were performed at the IN13 backscattering spectrometer at the ILL^16,17^ with λ = 2.23 Å. IN13 offers an energy resolution of δℏω ≈ 8 μeV and covers a large *Q* range.^18^ The neat protonated samples and the blends of both isotopic labels were investigated. The thicknesses of the samples were chosen such that a transmission of about 90% was expected. They were filling flat aluminum sample holders and placed at 135° with respect to the incident beam. The experiments consisted of recording the elastically scattered intensity in isothermal conditions for the different scattering angles, covering the effective *Q* range 0.52 ≤ *Q* ≤ 4.5 Å^–1^. At every temperature considered, the measuring time was of about 2 h. The samples were first cooled to 20 K, where the reference measurement was performed. Thereafter, measurements were performed in the temperature interval 50 ≤ *T* ≤ 300 K, with steps of 50 or 20 K (glassy state) and 10 K (around and above the calorimetric *T*_g_’s). The perpendicular transmission of the samples was determined to properly subtract the background signal measured on an empty cell at 285 K. The results at each temperature were normalized to the reference measurement at 20 K.

### Diffraction with Polarization Analysis

Exploiting polarization analysis, experiments by the D7 instrument^19^ at the ILL allowed accessing the ratio between coherent and incoherent differential scattering cross sections of the samples with 80 and 50% concentration of SBR measured at IN13. With λ = 4.88 Å a *Q* range from 0.13 to 2.46 Å^–1^ was covered. Experiments were performed at 300 K. The raw data were corrected for detector efficiency, flipping ratio, sample container, and absorption.

### X-ray Diffraction (XRD)

XRD experiments were performed in a Rigaku 3-pinhole PSAXS-L equipment at the Materials Physics Center in San Sebastián, Spain, using Cu Kα transition photons of λ = 1.54 Å. The two-dimensional multiwire X-ray detector (Gabriel design, 2D-200X) is a gas-filled proportional type detector offering a 200 mm diameter active area with ca. 200 μm resolution. After azimuthal integration, the scattered intensities were obtained as a function of *Q* in the range between 0.1 and 1.6 Å^–1^. Samples were placed in transmission geometry, and experiments were performed at RT. The magnitudes measured by the scattering techniques employed in this work on the blend samples are presented in the Supporting Information. XRD results are also shown in the Supporting Information.

## Results and Data Analysis

### SANS: Thermally Driven Concentration Fluctuations

**Figure 1:**
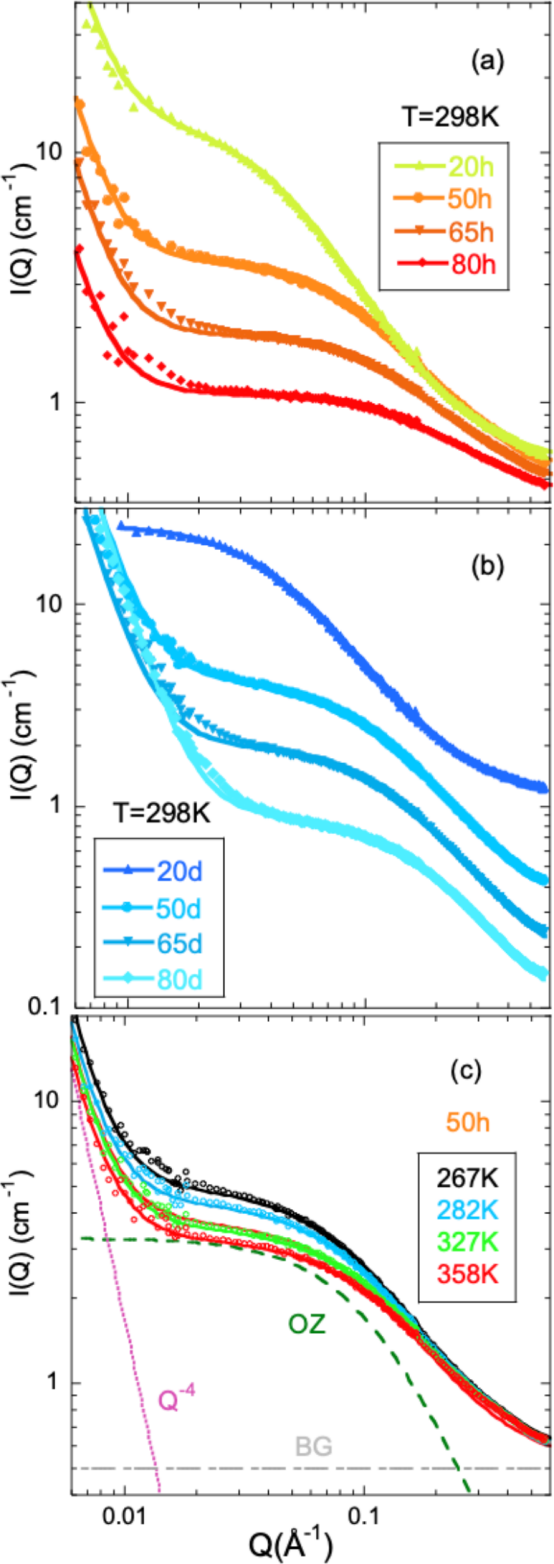
SANS results on (a) hSBR/dPS and (b) dSBR/hPS blends at 298 K and the compositions indicated. (c) Results on the hSBR/dPS blend with 50% composition at different temperatures. Solid lines are fits of eq 2; for the highest temperature, the fit components are shown (dotted line: power law ∝ *Q*^–4^; dashed line: Ornstein–Zernike; dashed-dotted line: flat background).

Representative SANS results are shown in Figure 1. With decreasing *Q*, the data show a first clear increase followed by a plateau. This regime is dominated by TCF in the mixture. The amplitude of this contribution strongly increases with PS concentration. For a given sample, as shown for the 50 h blend in Figure 1c, the amplitude of TCF increases with decreasing temperature. To characterize the TCF, the Ornstein–Zernike (OZ) expression is usually invoked:
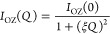
1where *I*_OZ_(0)—the *Q* → 0 value of the function—is the amplitude and ξ is the correlation length for concentration fluctuations. The OZ function is in general a good approximation of the structure factor of polymer blends in the random phase approximation (RPA).^20−24^ We note that applying the habitual RPA framework in the present system is not trivial because the Debye function is not the most appropriate functional form to describe the form factor of the oligomers. Therefore, the OZ function has been chosen. Below *Q* ≈ 0.015 Å^–1^, an additional contribution to the scattered intensity is found which varies as ∝ *Q*^–*x*^ with *x* ≈ 4. This kind of behavior was also presented by SBR/PS blends with higher molecular weight PS^13^ as well as in other dynamically asymmetric mixtures as e.g. blends of PS and poly(vinyl methyl ether) (PVME)^25,26^ or poly(ethylene oxide) (PEO) and poly(methyl methacrylate) (PMMA).^27^ The origin of this contribution to the scattering is controversial.^13,25−27^ It has been tentatively attributed to well-defined or “sharp” boundaries due to the presence of large domains,^25^ to excess inhomogeneity resulting from stress–diffusion coupling during temperature change,^26^ or to pronounced long-range density fluctuations.^27^ Its interpretation is beyond the scope of our work; in addition, without a model, it is impossible to extract any relevant length scale for this feature. Therefore, we have just parametrized it with a Porod-like power law ∝ *Q*^–4^ added to the OZ. A background (BG) is also considered to describe the SANS results:
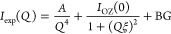
2The good quality of this kind of description can be appreciated in Figure 1. For the highest temperature, Figure 1c shows the separate contributions involved.

**Figure 2:**
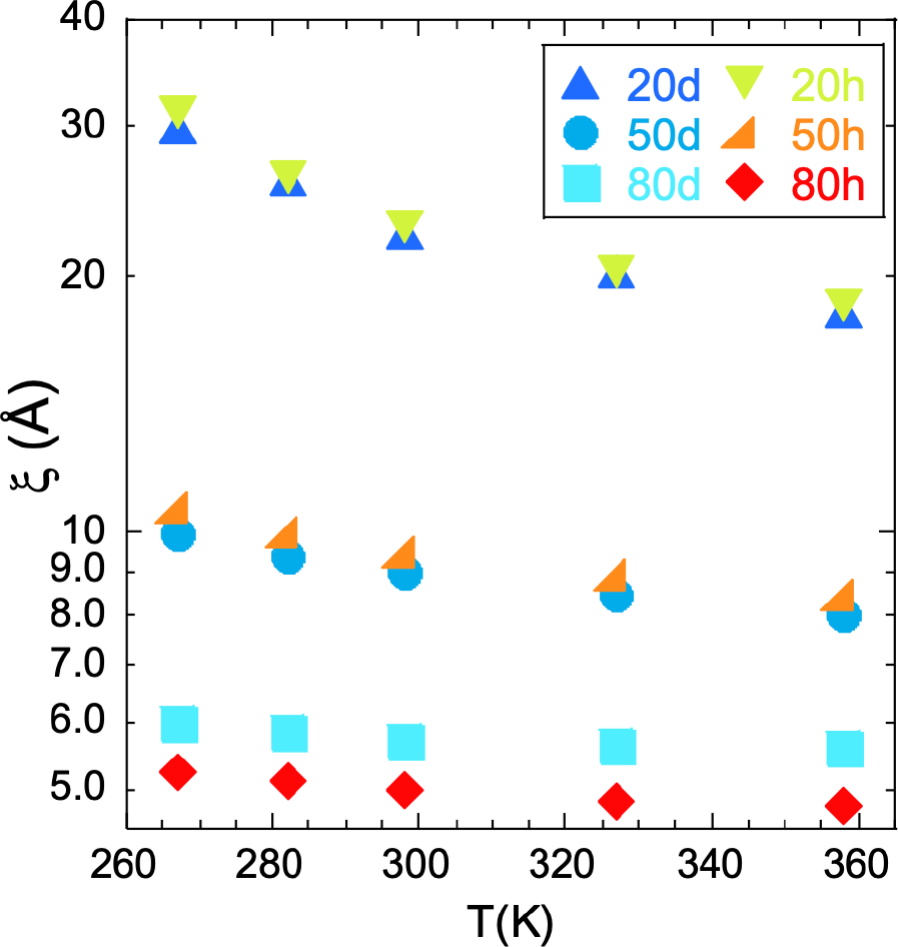
Temperature dependence of the correlation length for thermally driven concentration fluctuations.

The correlation length ξ is rather small and does not appreciably and systematically depend on the isotopic label ; at RT ξ ≈ 5–7 Å in SBR-rich blends, 8–9 Å in 50/50 blends, and around 20 Å in PS-rich mixtures. As can be seen in Figure 2, it increases with decreasing temperature, this tendency being particularly strong in the samples with highest concentration of PS. The inverse values of the OZ amplitudes *I*_OZ_(0) follow well a linear dependence as a function of the inverse temperature (see Figure S3.1). The amplification of the concentration fluctuations and the increase of the associated correlation length with decreasing temperature point to phase separation of the mixtures at low temperatures (upper critical solution temperature (UCST)-type phase behavior). The spinodal decomposition temperature *T*_s_ was determined to be the value at which *I*_OZ_(0) tends to diverge by assuming an extrapolation as *I*_OZ_^–1^(0) ∝ 1/*T* (see Figure S3.1). The resulting values are compiled in Table 4 together with calorimetric results (see later). The SANS experiments also allow determining the Flory interaction parameter between the two components, as shown in the Supporting Information.

**Table 4:** Spinodal Decomposition Temperature Obtained from SANS and Average, Initial, and Final Temperatures and Widths of the Calorimetric Glass Transitions Obtained from DSC

sample	*T*_s_ (K)	*T*_g_ (K)	*T*_g,init_ (K)	*T*_g,fin_ (K)	Δ*T*_g_ (K)
hSBR		207.0	202.4	211.3	9
80h	118.6	216.1	210.2	221.3	11
50h	188.5	229.8	221.4	238.0	17
20h	235.5	239.4	229.8	249.3	19
dPS		249.8	243.8	255.8	12
dSBR		208.8	203.5	213.9	10
80d	82.5	216.5	210.2	222.9	13
50d	178.3	228.6	219.2	237.7	19
20d	232.3	252.2	241.8	264.2	22
hPS		269.6	264.1	274.1	10

From the insight into TCF by SANS, we can also deduce the mean-squared concentration fluctuation ⟨δφ^2^⟩ in a given sample volume. On the basis of previous works of Fischer et al.,^28,29^ Colby and Kumar et al.^30^ proposed the following expression for ⟨δφ^2^⟩ in an incompressible binary blend:
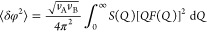
3where *v*_A_ and *v*_B_ are the monomeric volumes of the two species (see Table 2), *S*(*Q*) is the structure factor, and *F*(*Q*) is the form factor of the volume considered. Assuming for it a sphere of radius *R*_c_ and using the OZ approximation for the structure factor—which, as shown above, provides a very good description of the SANS data—eq 3 can be expressed as
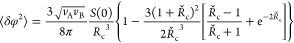
4Here, . Thus, using this expression,  can be calculated for different values of *R*_c_, with the input of the ξ and *S*(0) values deduced from the SANS experiments. The latter are obtained from the OZ amplitudes as *S*(0) = *I*_OZ_(0)/[*v*_o_(Δρ)^2^], where  and Δρ the difference in scattering length density. Some examples of σ^SANS^(*R*_c_) are shown in Figure S4.8.

### Elastic Fixed Window Scans: Microscopic Insight into Proton Displacements

**Figure 3:**
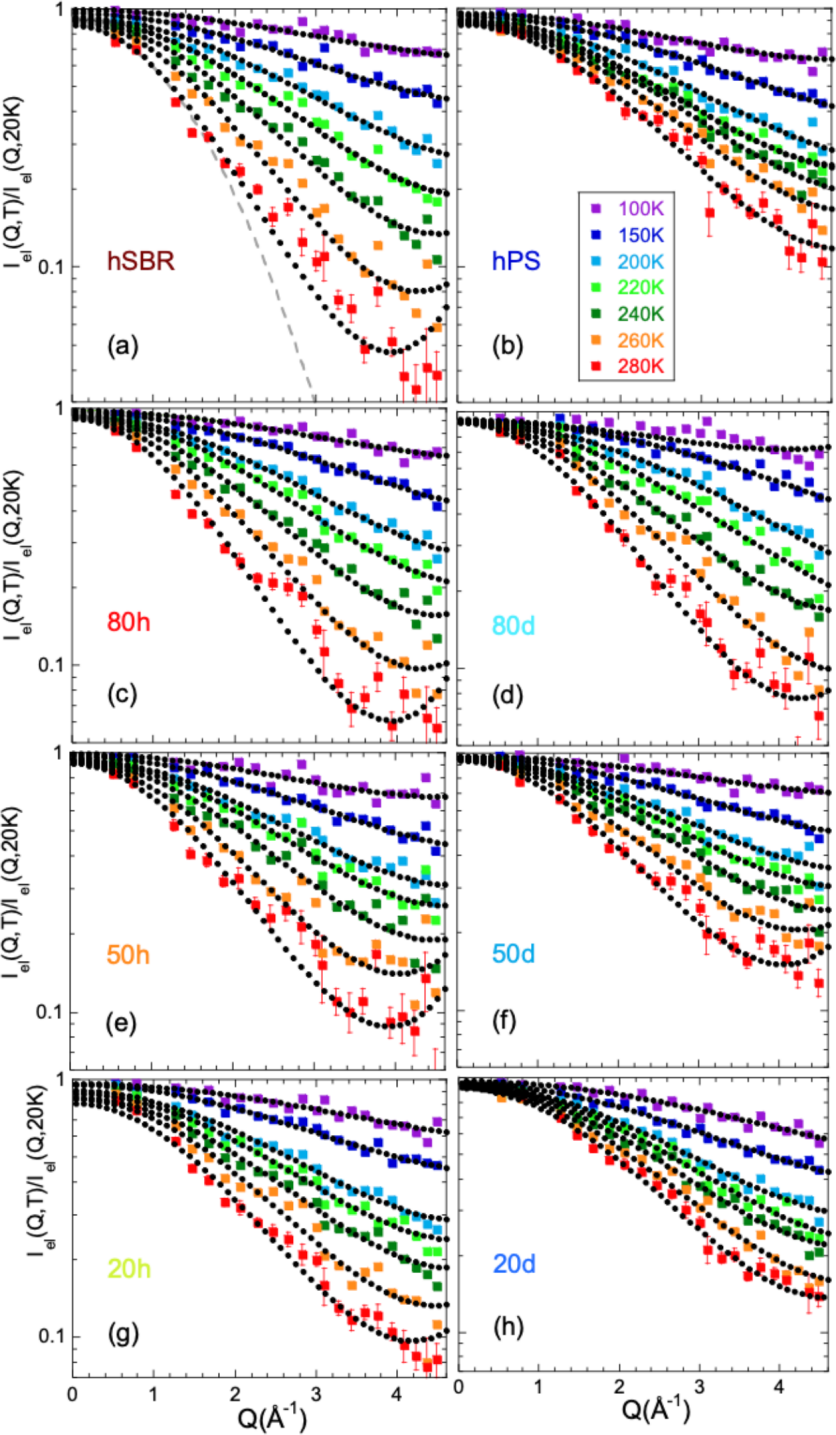
EFWS results obtained on the protonated neat components (hSBR (a) and hPS (b)) and on blends with decreasing SBR content (80% (c, d), 50% (e, f), and 20% (g, h)); panels on the left (a, c, e, g) correspond to hSBR/dPS samples, where the scattered intensity is dominated by the SBR component, and on the right to dSBR/hPS samples (b, d, f, h), where the results mainly reflect PS dynamics. Different colors correspond to different temperatures indicated in (b). Representative error bars are shown for the 280 K data. Dotted lines are fits of eq 6; the dashed line in (a) shows the description if only the leading term in eq 6 is considered.

**Figure 4:**
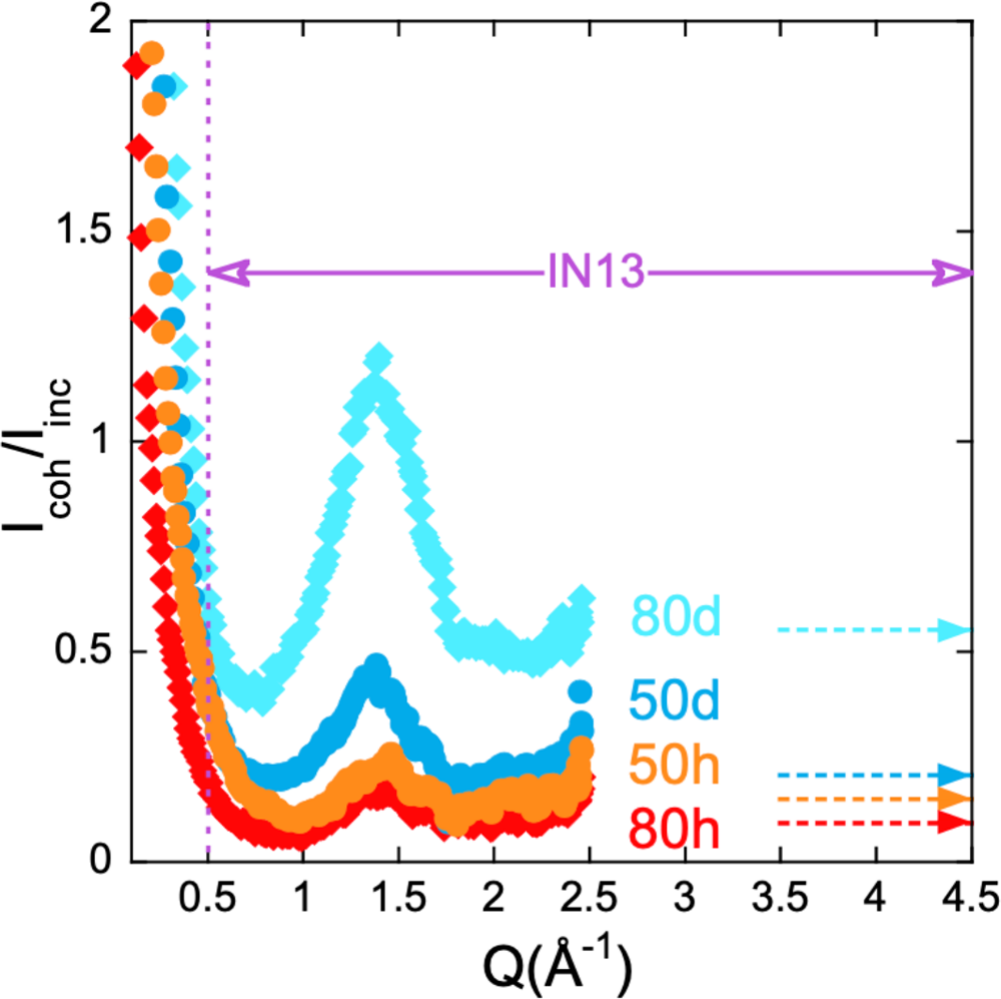
Ratio between the coherent and incoherent differential scattering cross sections determined by diffraction with polarization analysis (D7) on the blend samples investigated by IN13 with 80 and 50% SBR composition. The *Q* range covered by the IN13 experiments is marked by the horizontal solid arrow. Dotted horizontal arrows mark the theoretical value of the ratio between coherent and incoherent scattering cross sections σ_coh_/σ_inc_ for the different samples, which should be the high-*Q* asymptotic limit of the measured magnitude.

We now move to the component-selective and microscopic information offered by the EFWS experiments. Figure 3 shows the elastically scattered intensity recorded in the EFWS for the different samples investigated at selected temperatures, normalized by its value at a very low temperature (20 K). This magnitude decreases with increasing temperature and *Q*. As explained in the Supporting Information, the intensity scattered by our samples in the *Q* region explored by these experiments is predominantly of incoherent nature (see D7 results in Figure 4) and has its origin in the hydrogens. This means that for the homopolymers it reflects the atomic (H) displacements in the bulk system, while in the blends it selectively reveals those of the sample labeled with hydrogens. In particular, the results on the left panels of Figure 3 are sensitive to the hSBR component in the blends, while those on the right panels to the hPS component. Though not strictly exact,^31−34^ the EFWS results can be considered as an approximation to the incoherent intermediate scattering function *I*_inc_(*Q*,*t*) of the hydrogens in the sample at the instrumental resolution time *t*_R_ = *ℏ*/δℏω (see, e.g., refs (35−37)). In the IN13 configuration, δℏω ≈ 8 μeV and thus *t*_R_ ≈ 80 ps.

In general, *I*_inc_(*Q*,*t*) can be expressed as an expansion in *Q*^2^:
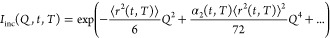
5Here the leading *Q*^2^ term is determined by the atomic mean-squared displacement ⟨*r*^2^(*t*)⟩—the second moment of the van Hove self-correlation function *G*_s_(*r*,*t*). The second term in the expansion accounts for deviations from the Gaussian form of *I*_inc_(*Q*,*t*) (equivalently, of *G*_s_(*r*,*t*)) through the second-order non-Gaussian parameter α_2_(*t*). α_2_(*t*) is defined in terms of the even moments of *G*_s_(*r*,*t*) as . ⟨*r*^2^(*t*)⟩ is the mean-squared displacement (MSD) of the atom. Usually a temperature-dependent prefactor *I*_o_ accounting for multiple scattering effects and normalization uncertainties^38−40^ has to be considered when dealing with experimental EFWS results; thus, assuming that the elastic intensity is an approximation of the scattering function at *t*_R_, the EFWS can be described by
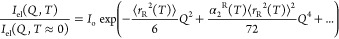
6where the effective MSD  and the effective non-Gaussian parameter . The first term of the expansion is usually enough to describe the EFWS results for small *Q* values. In a glassy solid the ⟨*r*^2^(*t*_R_)⟩ can be identified with the average atomic (H) displacements within the cage imposed by the neighbors. However, at high temperatures the meaning of the effective MSD has to be cautiously considered. When quasi-elastic contributions become of the same order as the instrumental resolution the ⟨*r*_R_^2^⟩ values are affected by them. In fact, they depend on the considered instrumental resolution (time-dependent MSD). In general, the effective MSD results reflect the decay of *I*_inc_(*Q*,*t*) through fast processes (vibrations and rapid motions) and also relaxational processes.

**Figure 5:**
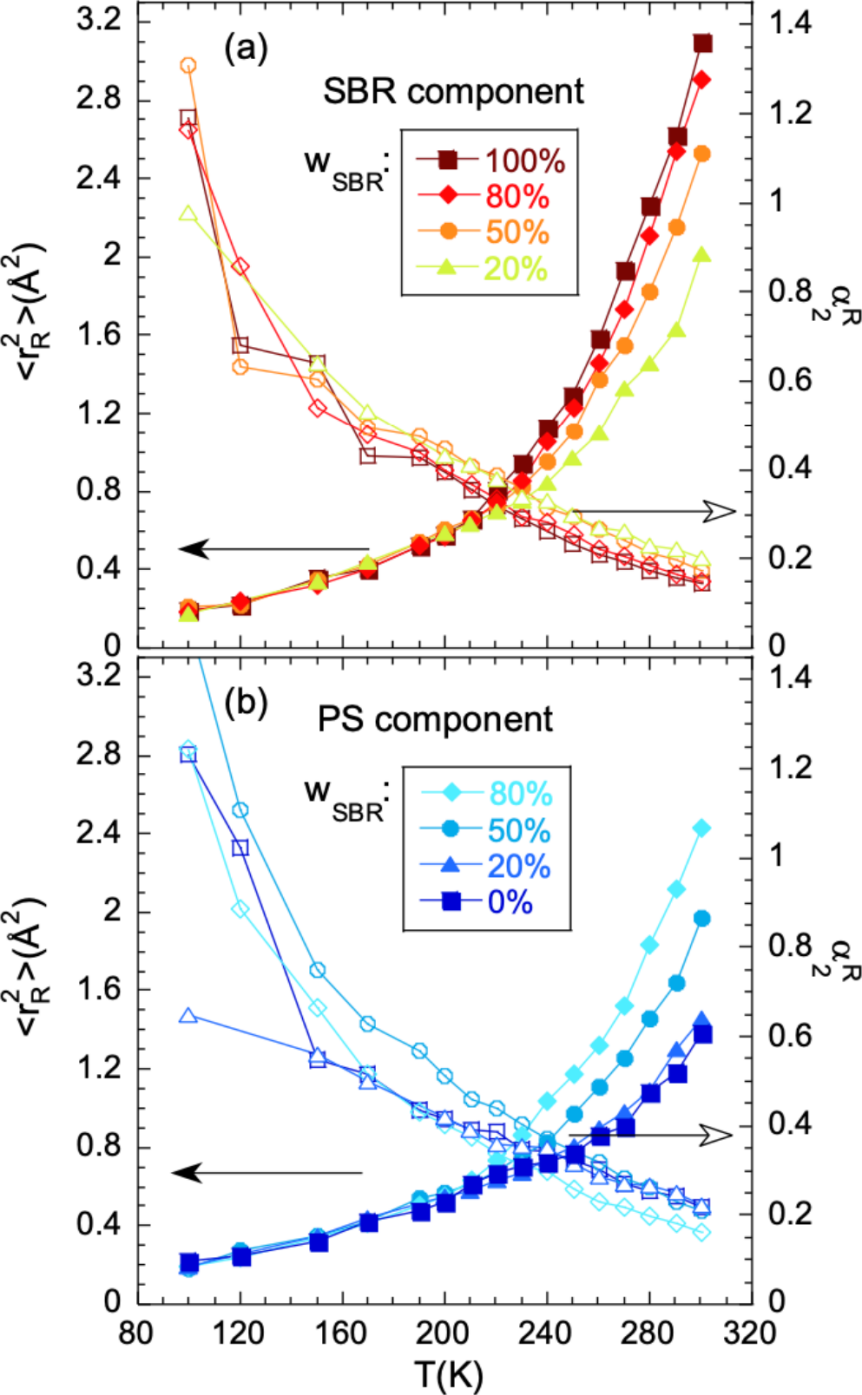
Effective mean-squared proton displacement (filled symbols, scale on the left) and effective non-Gaussian parameter (empty symbols, scale on the right) deduced from the fits of eq 6 to the IN13 results on the hSBR/dPS samples (a) and on the dSBR/hPS samples (b). Different symbols correspond to different SBR compositions indicated; lines connecting points are guides for the eye.

We fitted eq 6 to the EFWS results (see Figure 3). As shown in panel a for the example of pure hSBR, the huge *Q* range covered by IN13 clearly demands for the use of the second term in eq 6 accounting for deviations from Gaussian behavior. Very good descriptions of the experimental data were obtained in this way. The resulting values of the fitting parameters ⟨*r*_R_^2^⟩ and α_2_^R^ are displayed in Figure 5. Panel a shows the results for neat hSBR and the hSBR component in the blends, and panel b shows those on hPS and the hPS component in the mixtures. In all cases the effective MSD ⟨*r*_R_^2^⟩ increases with temperature, while the α_2_^R^ values reflecting deviations from the Gaussian behavior decrease. At low temperatures, in the glassy state, all results for ⟨*r*_R_^2^⟩ are practically identical, within the uncertainties, to those of the neat polymer. This could be expected because blending does not appreciably affect dynamical processes involved in the glassy state, like vibrations or secondary relaxations.^2^ At a given temperature that depends on composition, the effective displacements within the mixtures start to differ from those in the homopolymer. In the high-temperature region, the SBR ⟨*r*_R_^2^⟩ decreases with increasing PS content in the blend, while the PS effective displacements increase with increasing amount of surrounding SBR.

### Calorimetric Results: Component Contributions to the Specific Heat

The glass transition manifests as a step in the specific heat. Even in homopolymers, this process usually extends over a given temperature range, and therefore to properly characterize it, not only the average value of the glass transition temperature has to be determined but also its width.^41^ The average *T*_g_ value is usually determined from the inflection point of the spectific heat *C*_*p*_ (correspondingly, from the position of the maximum in the *T* derivative of *C*_*p*_). The initial (*T*_g,init_) and final (*T*_g,fin_) transition temperatures are representative for the temperatures where *C*_*p*_ departs from the glassy and “equilibrium” supercooled-liquid behavior, respectively. They are determined using constructions as that illustrated in Figure S1. The width of the glass transition is defined as Δ*T*_g_ = *T*_g,fin_ – *T*_g,init_. The values of the temperatures characterizing the glass-transition processes in the different systems investigated are listed in Table 4. The value of *T*_g_ is always higher than *T*_s_: upon cooling, the sample becomes a glass before demixing.

The SBR homopolymers present similar *T*_g_ and Δ*T*_g_ values for both isotopic labels: *T*_g_ = 207 K (hSBR) and 209 K (dSBR); Δ*T*_g_ = 9 K (hSBR) and 10 K (dSBR). In the PS samples the *T*_g_ values differ more: *T*_g_ = 250 K (dPS) and 270 K (hPS), with Δ*T*_g_ = 12 and 10 K, respectively. Isotopic effect may be one the reasons for the difference in *T*_g_ values; however, the main origin in the PS case can be attributed to the difference in the molecular size of these oligomers. From the *M*_w_ values we can infer that in average the hydrogenated molecule has six phenyl rings while the deuterated one has five. On the other hand, the *T*_g_ values of the SBR and PS homopolymers used for the blends differ each other by 43 K (hSBR/dPS blends) and 61 K (dSBR/hPS blends), allowing to categorize the mixtures as dynamically asymmetric. The *T*_g_ and Δ*T*_g_ values in the blends increase with PS content (see Table 4). The width of the glass transition is not symmetric with composition: while the PS-rich blends show a very broad glass-transition process, the DSC traces in SBR-rich mixtures are not significantly broadened with respect to SBR homopolymers.

**Figure 6:**
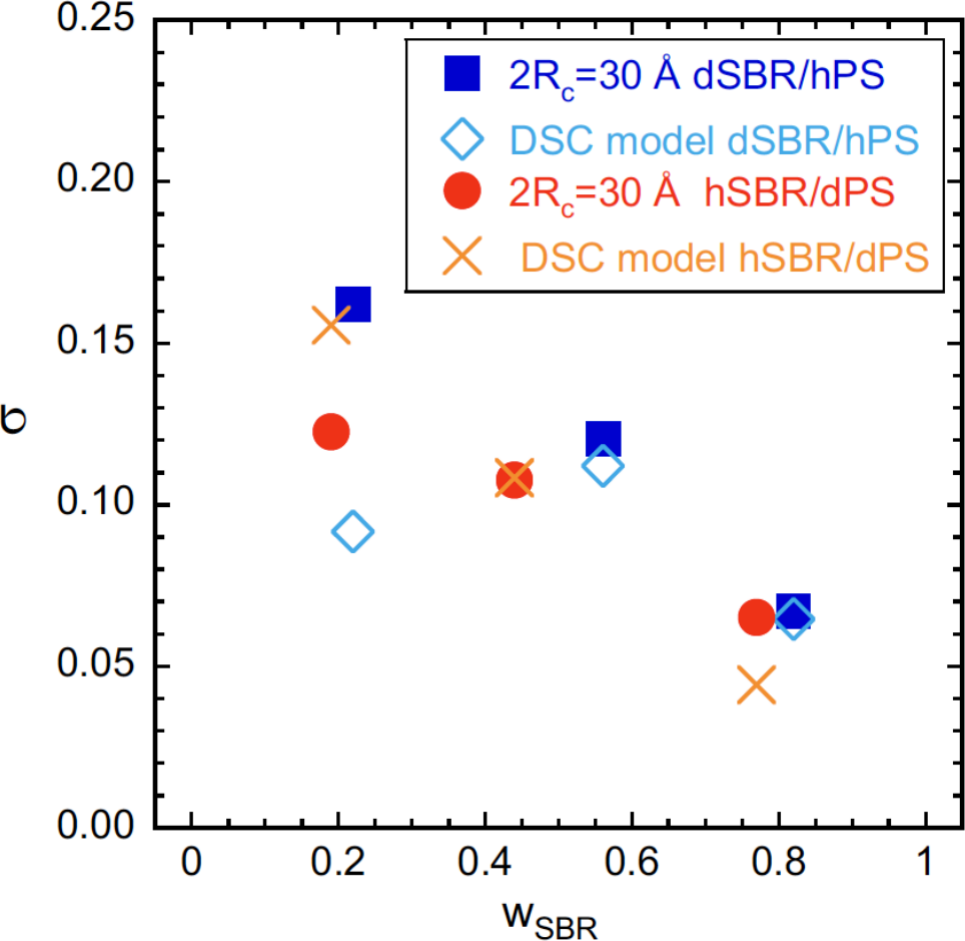
Composition dependence of the standard deviation of the distribution of concentration obtained from the DSC analysis (crosses: hSBR/dPS blends; diamonds: dSBR/hPS blends) and deduced from SANS results for a relevant length scale of 3 nm (filled circles: hSBR/dPS blends; filled squares: dSBR/hPS blends).

**Figure 7:**
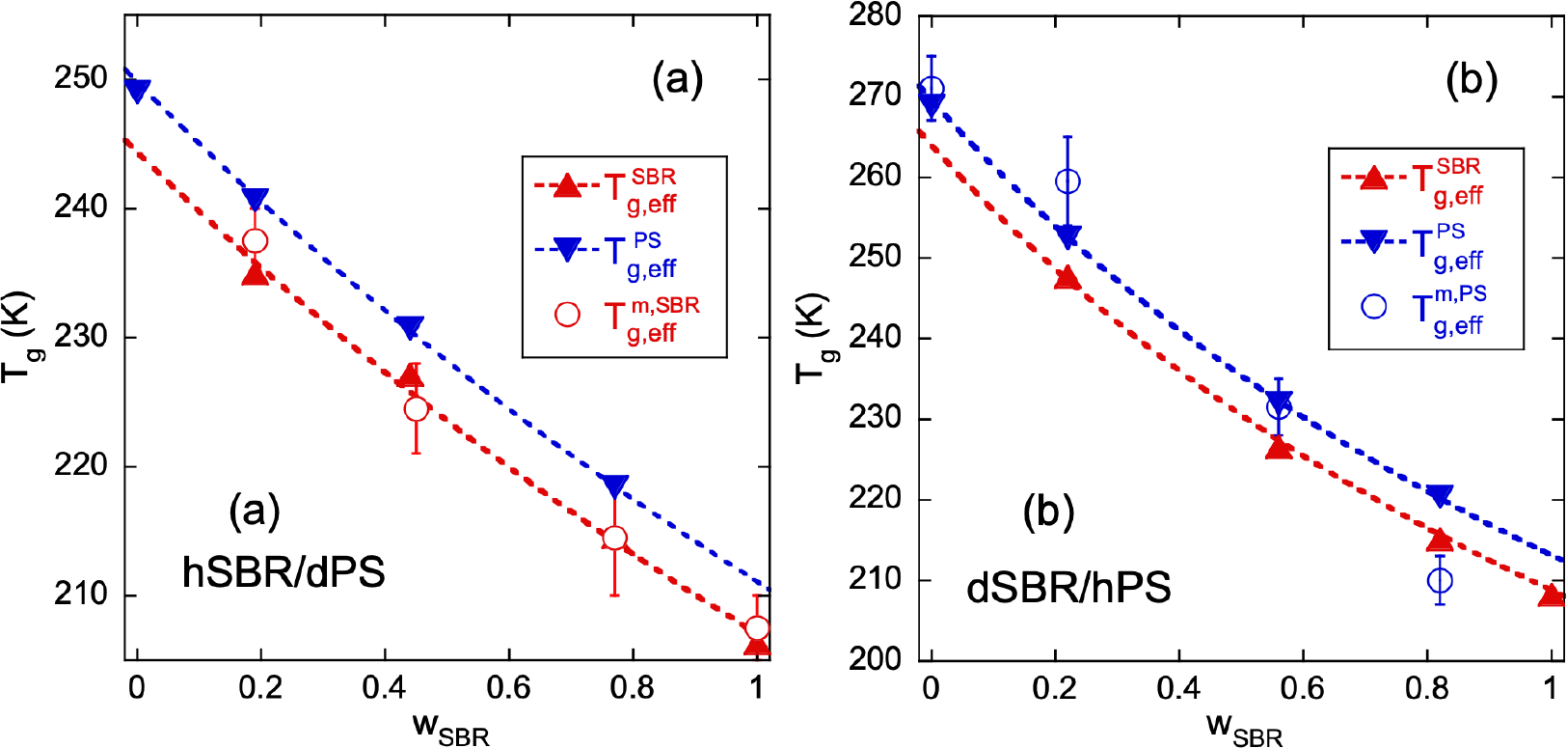
Composition dependence of the effective glass-transition temperatures identified on the systems based on hSBR and dPS (a) and on dSBR and hPS (b). Triangles represent the effective glass-transition temperatures obtained from the application of the model to the DSC results (up-triangle: SBR component; down-triangle: PS component). Dotted lines are the Gordon and Taylor equations accounting for self-concentration effects (see the Supporting Information). The values of the microscopic effective glass-transition temperature *T*_g,eff_^m^ determined from EFWS for the protonated component are represented by the circles. Error bars in these results arise from the uncertainties in their determination.

We disentangled the two contributions to the DSC glass-transition process, corresponding to each of the components, applying the model mentioned in the Introduction to the present case. The bases of the model and the procedure followed in its application are explained in detail in the Supporting Information. Here we just mention that it assumes a quasi-static Gaussian distribution of concentrations centered around the bulk composition of the blend, arising from TCF, and also implements the ingredient of the “self-concentration”. The description obtained for the DSC response is excellent, as can be appreciated in Figure S4.7. The only fitting parameters used were the self-concentration φ_self_ of the two components, accounting for SC effects, and the standard deviation of the TCF σ. The values for φ_self_ were assumed to be independent of temperature, concentration, and isotopic labeling, obtaining φ_self_^SBR^ = 0.03 and φ_self_^PS^ = 0.21. The values of σ were assumed to be independent of temperature. They turn to be concentration-dependent and are plotted in Figure 6. The deduced values of the effective glass-transition temperatures of the two blend components are listed in Table 5 and represented as triangles in Figure 7.

**Table 5:** Glass Transition Temperature and Effective Glass-Transition Temperatures of the Two Components Obtained from the Application of the Model and Microscopic Glass-Transition Temperature Determined from the EFWS

sample	*T*_g_ (K)	*T*_g,eff_^SBR^ (K)	*T*_g,eff_^PS^ (K)	*T*_g,*eff*_^m,SBR^ (K)	*T*_g,eff_^m,PS^ (K)
hSBR	206.3			207.5 ± 2.5	
80h	214.9	214.6	218.4	214.5 ± 4.5	
50h	228.6	227.1	230.7	224.5 ± 3.5	
20h	238.7	235.0	240.7	237.5 ± 3.5	
dPS	249.0				
dSBR	208.2				
80d	215.6	215.1	220.4		210.0 ± 3.0
50d	228.2	226.6	232.0		231.5 ± 3.5
20d	251.0	247.7	252.5		259.5 ± 5.5
hPS	268.8				271.0 ± 4.0

## Discussion

### Microscopic Trace of the Glass Transition

**Figure 8:**
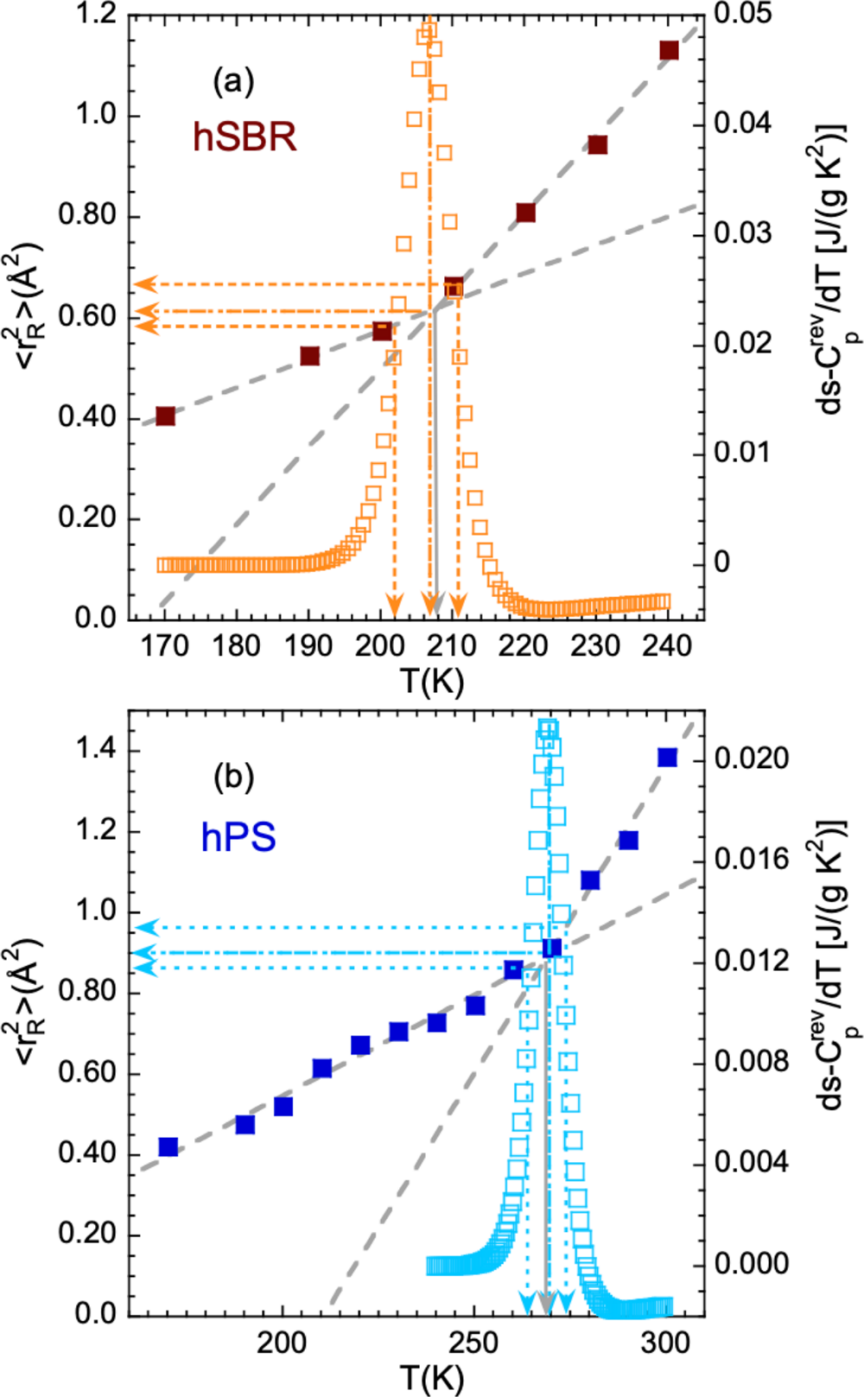
Effective mean-squared proton displacement (filled symbols, scale on the left) and temperature derivative of the segmental part of the reversible heat flow from DSC (empty symbols, scale on the right) corresponding to the neat protonated systems: hSBR (a) and hPS (b). The dashed lines represent the linear dependence of ⟨*r*_R_^2^⟩ in the glassy state above 150 K (⟨*r*_R,g_^2^⟩) and in the supercooled state (between *T*_g_ and *T*_g_ + 30 K approximately). From their crossing, the *T*_g_^m^ value is obtained (marked by the solid arrow). The vertical arrows show the average (dashed-dotted), initial, and final (dotted) calorimetric glass-transition temperatures, and the corresponding horizontal arrows mark the values of ⟨*r*_R_^2^⟩ at these temperatures.

The microscopic insight into the hydrogen displacements provided by the EFWS can be compared with the results of the “macroscopic” DSC technique. This is done in Figure 8 for the two homopolymers. In both cases, the hydrogen ⟨*r*_R_^2^⟩ follows well a linear temperature dependence in the glassy state above 150 K: ⟨*r*_R,g_^2^⟩ (Å^2^) = −0.551 + 0.00563*T* [K] for hSBR and ⟨*r*_R,g_^2^⟩ (Å^2^) = −0.450 + 0.00498*T* [K] for hPS. At a given temperature, additional contributions to the extrapolated low-*T* linear behavior can clearly be detected in ⟨*r*_R_^2^⟩. As can be appreciated in Figure 8, the onset temperature of these contributions (solid arrow) is located in the neighborhood of the calorimetric glass transition (dashed-dotted arrow). With these results we corroborate in our homopolymers such a commonly found coincidence (see, e.g., refs (7−10)). The MSD at times of the order of tens of picoseconds thus constitutes a sensitive probe to detect motions involved in the supercooled liquid regime, when the dynamic arrest induced in the glassy state is released. This microscopic magnitude clearly reveals at which temperature the environment of H nuclei softens enough for accommodating atomic displacements characteristic for the supercooled liquid that are not allowed in a frozen medium. We can thus identify this temperature with the “microscopic” *T*_g_ in the system and shall denote it as *T*_g_^m^. For hSBR *T*_g_^m^ = 207.5 ± 2.5 K, and for hPS *T*_g_^m^ = 271.0 ± 4 K.

A connection between the α-relaxation process—with associated characteristic times of the order of seconds in the vicinity of the glass transition—and mean-squared displacements in the picosecond–nanosecond time scale is apparently surprising, but it has been repeatedly reported in the literature; a seminal work in this direction is the study on selenium by Buchenau and Zorn.^42^ However, we note that while the α-process is slow, the barrier transitions underlying this relaxation are fast. This is at the basis of the so-called elastic models.^11,12^

It has been suggested that the well-known Lindemann criterion that applies for crystalline systems can be extended to inhomogeneous systems^43^ and even proteins.^44,45^ A Lindemann-like criterion for the glass transition can be deduced in a straightforward way in the framework of elastic models,^11^ and also invoking different arguments (see, e.g., refs (46 and 47)). We recall that the Lindemann criterion predicts the melting of crystals on the basis of the relative magnitude of thermal atomic fluctuations and the crystal lattice constant. When this magnitude exceeds about 0.1–0.2, melting occurs. The analogous parameter in glass-forming systems related to the glass-transition phenomenon (Δ_Lg_) would be the ratio between the root of the mean-squared fluctuation MSF at *T*_g_ and the average intermolecular distance *d*_chain_. The MSF in the harmonic approximation MSD = 2MSF, and  would be an estimation of the Lindemann parameter. Taking into account the values of the effective MSD at the glass-transition temperature (see Figure 8) and the values of *d*_chain_ determined from the X-ray diffraction experiments (*d*_chain_ = 4.7 Å for SBR and 4.8 Å for PS; see the Supporting Information), we can determine  = 0.12 for hSBR and 0.14 for hPS. Thus, the value for hSBR is slightly smaller than for hPS, and both are in the range reported for crystals, as mentioned above, as well as for the melting of proteins.^45^ Our findings would also support the results obtained by Leporini et al.^48^ However, the existence of a universal Lindemann criterion as predicted in ref (48) was not confirmed in some molecular liquids.^8^ Interestingly, by investigating in ref (8) the MSD at various temperatures and pressures for a number of molecular glass-forming liquids, an intrinsic Lindemann criterion was found for any given liquid. The existence of an intrinsic Lindemann criterion is in fact predicted by the above-mentioned elastic models.^11,12^

**Figure 9:**
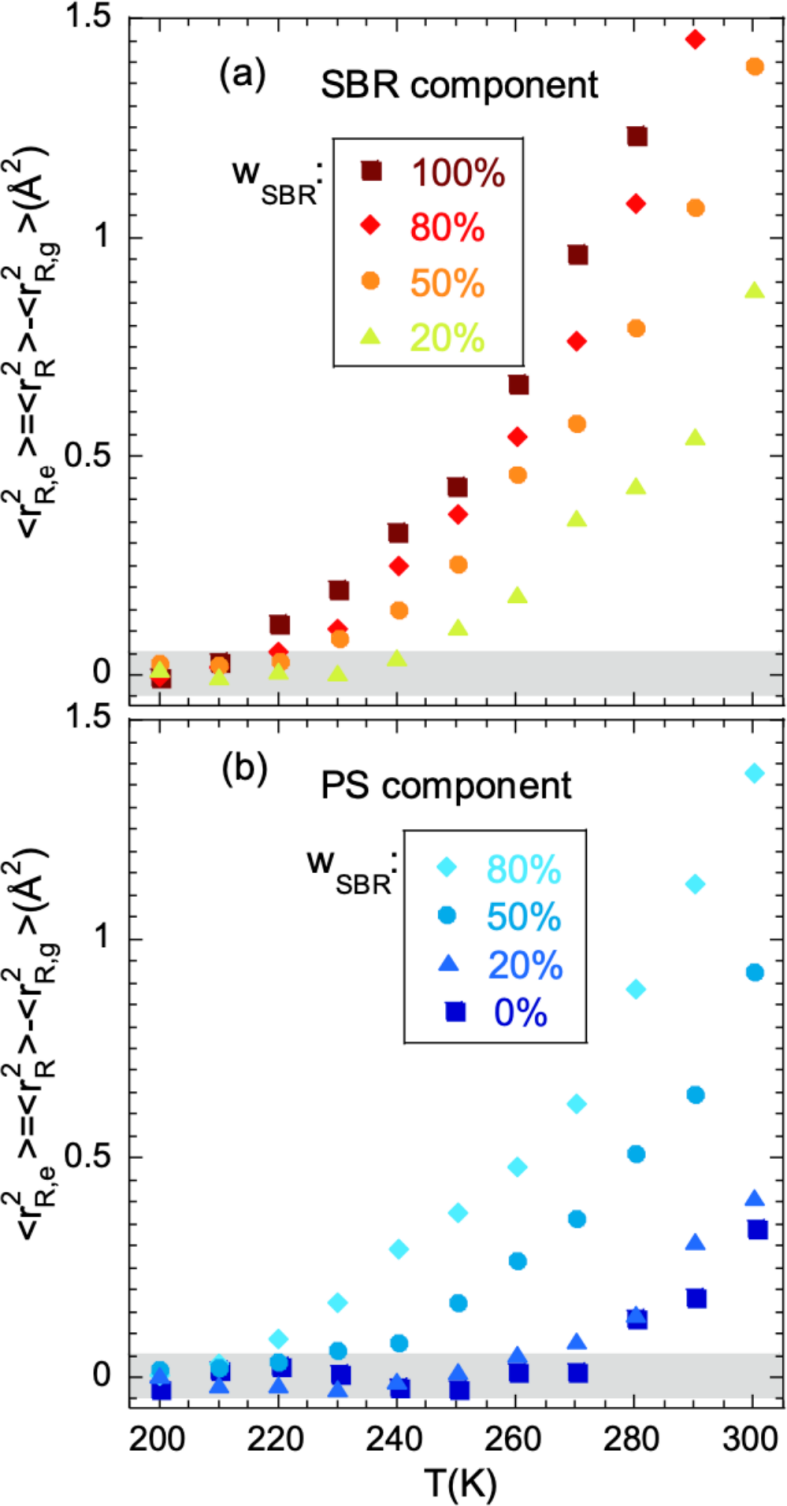
Temperature dependence of the “excess MSD”, defined as the difference between ⟨*r*_R_^2^⟩ and the expected “glassy” value of this magnitude for the corresponding neat component (linear law fitting of  in the range 150 K ≤ *T* ≤ *T*_g_, dashed lines in Figure 8). Panel a shows the results on the hSBR/dPS blends, and panel b shows the results on the dSBR/hPS mixtures. The dashed area shows representative uncertainty in the reference level.

The atomic displacement at times of some tens of picoseconds can thus be considered as an important magnitude related to the glass-transition phenomenon in the material, even in complex systems like polymers. Let us now consider the more complicated case of the blends. As can be appreciated in Figure 5, in the blends a qualitatively similar behavior of ⟨*r*_R_^2^⟩ as for the homopolymers is found, though the onset of additional displacements with respect to the glassy behavior occurs at a different temperature. In analogy with the homopolymers, and taking into account the selectivity of the EFWS experiments to the protonated component in the mixture, we can identify this temperature as the “microscopic *T*_g_” of the labeled component in the blend; in the terminology used for macroscopic results as from BDS and DSC, it would thus be the “effective microscopic” glass-transition temperature *T*_g,eff_^m^. For a quantitative analysis of the EFWS results, we have calculated the “excess in effective mean-squared displacement” with respect to the expected value in the glassy state (). The latter has been assumed to be independent of composition and equal to that previously determined for the corresponding neat polymer—we recall that we observed indistinguishable effective MSD, within the uncertainities, in the glassy state for all samples of a given family (see Figure 5). The results for ⟨*r*_R,e_^2^⟩ are shown in Figure 9. From this representation we have determined the effective microscopic glass transition for both blend components at the different concentrations investigated. In this estimation, the uncertainties associated with the extrapolation of the low-temperature value (gray areas in Figure 9) and initial slope of the high-*T* behavior have been considered. This leads to define intervals of temperatures within which the microscopic *T*_g_s would be located. We have then taken the middle of the interval as the corresponding microscopic *T*_g_ values. The results are listed in Table 5 and represented in Figure 7 together with the calorimetric results. *T*_g,eff_^m,SBR^ determined for the hSBR component from the EFWS coincides, within the uncertainties, with the “macroscopic” effective *T*_g,eff_^SBR^ deduced by the application of the model to the DSC results (see Figure 7a). In the case of the dSBR/hPS mixtures, where *T*_g,eff_^m^ corresponds to the onset of PS liquid-like displacements, its location seems to be close to the “macroscopic effective *T*_g,eff_” of the PS component for medium-high PS content and close to the “macroscopic effective *T*_g*,*eff_” of the dSBR component for the highest dSBR content (see Figure 7b). Thus, for the samples with medium or rich content in PS we find that the microscopic and macroscopic effective glass-transition temperatures coincide for PS as well as for SBR. In a given blend, these temperatures are different , and thus we deduce that the dynamics at the microscopic level is heterogeneous. Conversely, for the sample where SBR is the majority component, *T*_g,eff_^m^ of PS is close to *T*_g,eff_ of dSBR (see Figure 7b). This temperature is expected to coincide with *T*_g,eff_^m^ of dSBR (as it does for the opposite labeling, Figure 7a). In the case of the SBR-rich (80%) sample, thus, we find a homogeneous dynamic behavior at microscopic level. We note that in these conditions *T*_g,eff_^hPS^ is higher than its microscopic counterpart, implying that the loss of equilibrium observed by DSC occurs at higher temperatures. Between these two temperatures, i.e., *T*_g,eff_^m,PS^ ≈ *T*_g,eff_^SBR^ < *T* < *T*_g,eff_^PS^, the system is “liquid-like” at microscopic level but the PS component is “glassy-like” at macroscopic level.

In a previous work,^3^ we applied EFWS to isotopically labeled samples of SBR and PS oligomers with 900 kg/mol with 50/50 composition. We found out that the signature of microscopic glass transition occurred when the (broadened) distribution of macroscopic effective glass transition temperatures of the tagetted component started to present significant values. Those results support our present finding on the intermediate concentration samples. In a recent work^49^ on a mixture of a high-*T*_g_ resin with SBR (being SBR the majority component), we noted a coincidence of the microscopic glass transition determined from EFWS for the resin with the overall calorimetric *T*_g_ of the blend. Though the model was not applied to the DSC data to determine the effective glass transitions, we can expect that this result implies that also in that mixture both components experience their microscopic transition at the same temperature, as we find in the present case for the SBR-rich blend. We also mention a previous related work by Alba-Simionesco et al.,^50^ where a comparison of DSC and neutron scattering results was performed on a blend of high- and low-molecular-weight PS chains. However, in that work the components’ responses were not determined individually.

The change from heterogeneous to homogeneous behavior as a function of composition would be expected, in principle, to be gradual. Exploring intermediate concentrations in the medium to SBR-rich composition range would be interesting in order to characterize such a crossover and could be subject of future research.

**Figure 10:**
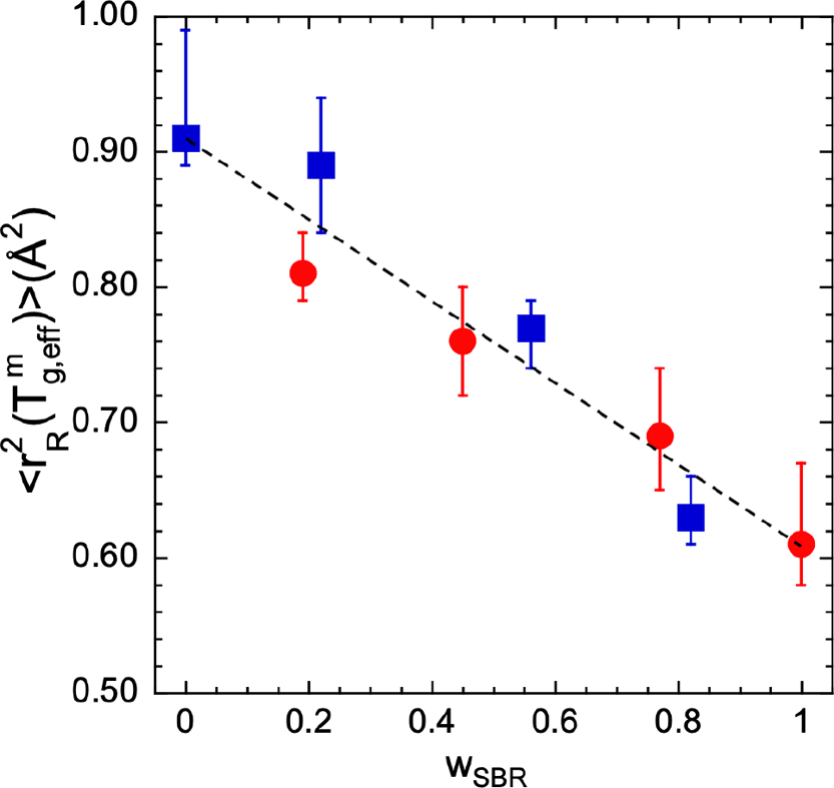
Composition dependence of the proton effective mean-squared displacement of hSBR (circles) and hPS (squares) at the corresponding “microscopic” effective glass transition deduced from the IN13 results.

Furthermore, we may ask how the Lindemann criterion applies in the blends. Figure 10 shows the effective MSD of each component in the blend at its “microscopic” effective glass transition. The ⟨*r*_R_^2^⟩ values shown in the blends by both PS and SBR are clearly different from the values observed when they are in their respective pure forms. This suggests that the miscibility of the components takes place at the molecular level. We can also say that within the uncertainties the displacements of both components when they feel the microscopic softening are very similar. This would allow us to propose a kind of mixing rule for the Lindemann criterion in the blends.

### “Microscopic” vs “Macroscopic” Self-Concentration

The value of the self-concentration for PS determined from the DSC analysis is φ_self_^PS^ = 0.21 independently of the isotopic label. For dPS900 oligomers of 900 g/mol, the value found was 0.19–0.20. Thus, there is no strong dependence of this magnitude on molecular weight. For the case of SBR, the value found for φ_self_^SBR^ = 0.03 is rather small, lower than those reported for other SBR components in this kind of blend: φ_self_^SBR^ = 0.14^5^ and φ_self_^SBR^ = 0.20.^4,13^ There is no clear correlation of φ_self_^SBR^ with its molecular weight (*M*_w_ = 10.6 kg/mol in ref (5); *M*_w_ = 23.5 kg/mol in refs (4) and (13)). A possible correlation could be found with the microstructure: the present samples, displaying a smaller value of φ_self_^SBR^, have a larger content in 1,4-butadiene units and a smaller content in styrene units.

From the EFWS we have deduced that the microscopic effective glass-transition temperature *T*_g,eff_^m^ of SBR coincides with the “macroscopic” one. Thus, for the fast component we observe a small “microscopic” self-concentration, similar to that “macroscopically” found. This finding provides additional support for the validity of the model used for the DSC analysis and corroborates the low value of φ_self_^SBR^. The much lower molecular weight of the polystyrene oligomer used in this work could be the reason for such a small value. Preliminary results obtained by mixing the same SBR with a higher-molecular-weight styrene oligomer point in this direction. The PS slow component shows a different behavior: for medium-high PS concentrations, it displays an apparently large “microscopic” self-concentration, similar to the macroscopic one. When it is the minority component—at least in the explored 80% SBR concentration—it “loses its identity” and becomes “animated” by the fast SBR majority component. For this composition, there is a significant difference between the value of ⟨*r*_R_^2^⟩ of this component at the temperature where it “feels” its microscopic glass transition  = 210 K) = 0.63 Å^2^) and the temperature where it “feels” its macroscopic glass transition  = 220 K) = 0.74 Å^2^).

### Non-Gaussian Effects and Their Origin

**Figure 11:**
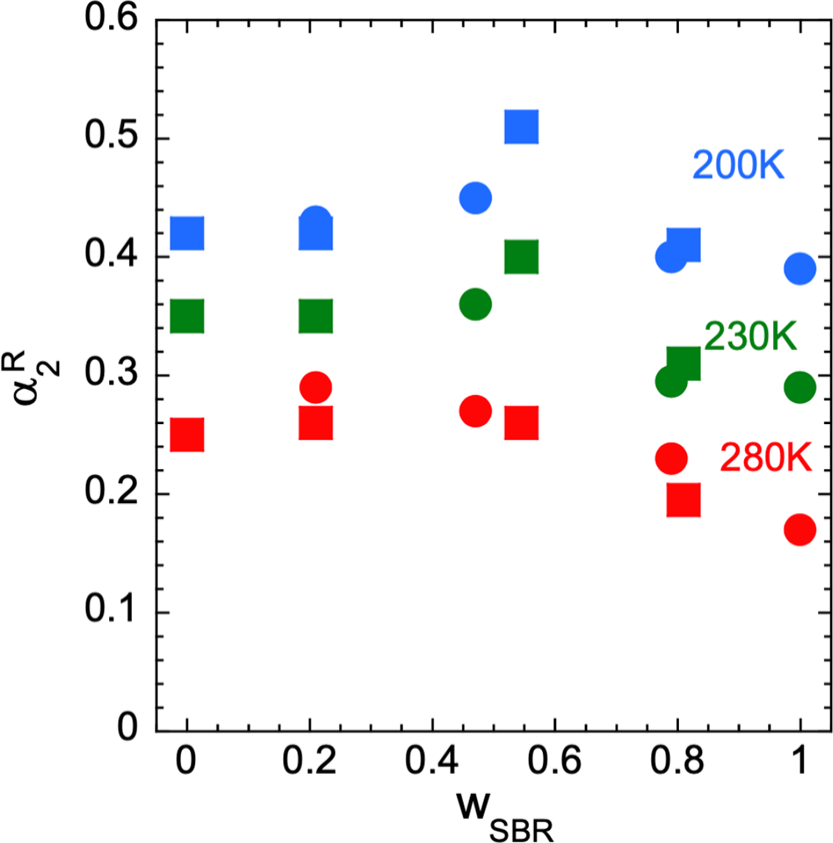
Composition dependence of the non-Gaussian parameter for three different temperatures. Circles correspond to SBR in hSBR/dPS samples and squares to PS in dSBR/hPS samples.

The values obtained for the α_2_^R^ parameter are shown as a function of temperature in Figure 5 and in Figure 11 as a function of composition (i) at 200 K, where all samples are in the glassy state, (ii) at 230 K, and (iii) at 280 K, all samples above their average macroscopic glass transition. As mentioned above,  if the normalized elastic intensity is a good approximation of the scattering function at the resolution time *t*_R_. The non-Gaussian parameter α_2_ accounts for deviation of atomic displacements from Gaussian behavior. The deviations reflected by this parameter can have diverse origins. First of all, they can be due to intrinsic heterogeneities associated with different motions in different locations of the polymer chain: main-chain versus side-group motions, end-chain additional fluctuations, particular dynamics at the different kinds of monomers (styrene, 1,4-butadiene, and 1,2-butadiene in the case of SBR, etc.);^51,52^ these effects are present both in the neat polymers and in the blends. They are expected to be less pronounced with increasing temperature—when usually characteristic times tend to converge—but would persist even at high temperatures. Second, as in any glass-forming system, non-Gaussian events associated with the cage dynamics, before the Gaussian subdiffusive regime is reached,^53^ contribute to these deviations. For homopolymers and other glass-forming liquids, MD simulations in a variety of systems^54−60^ show that the α_2_ parameter decreases with enhanced mobility (in particular, in relation to the dynamics of the α-relaxation). Thus, when the temperature increases, α_2_ decreases, indicating more Gaussian atomic displacements. This is the behavior found for the α_2_^R^ parameter in our systems (see Figures 5 and 11). Third, an additional source of contributions to deviations from Gaussian behavior, now specific for the polymeric mixtures, is the distribution of mobilities due to diverse environments associated with concentration fluctuations. We note the similar concentration dependence of α_2_^R^ and the standard deviation of the distribution of concentration determined from the SANS analysis (compare Figures 11 and 6). Also, as above argued from the comparison of the different effective glass-transition temperatures, from σ we can deduce a more heterogeneous microscopic behavior in the blend with high PS content than for the high SBR concentrations, in accordance with the tendency observed for α_2_^R^. Thus, even though the differences between the normalized elastic intensity and the actual scattering function at the resolution time *t*_R_ may lead to an apparent enhancement of non-Gaussian effects,^31^ the observed α_2_^R^ values show the trends expected for the underlying true non-Gaussian parameter.

### Relevant Length Scales in the Game

**Figure 12:**
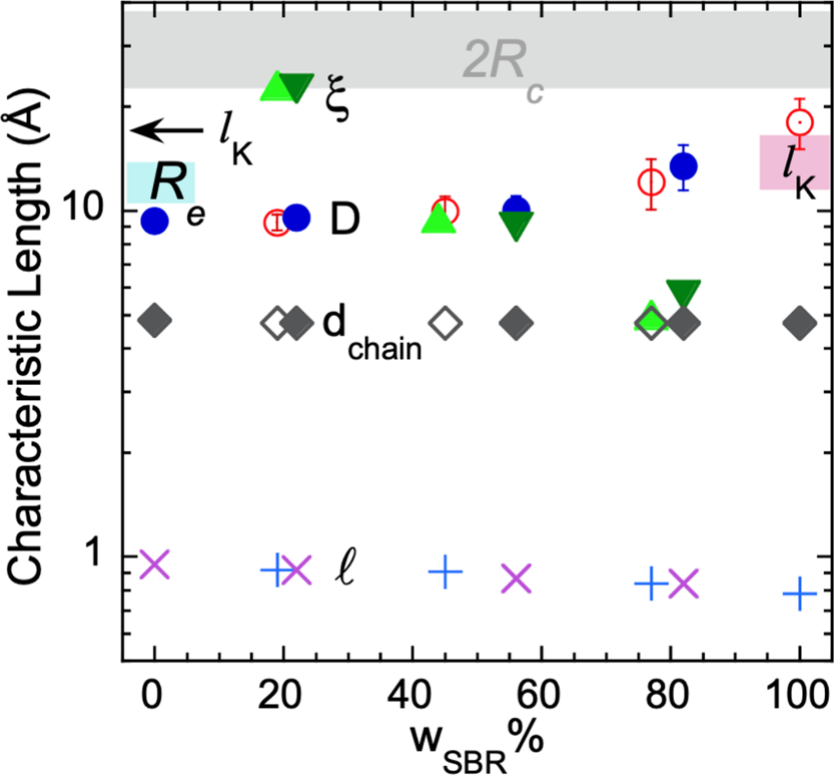
Composition dependence of the different characteristic length scales identified in this study: intermolecular distance *d*_chain_ (diamonds), inter-nanodomain distance *D* (circles) for hSBR/dPS (filled) and dSBR/hPS (empty) samples; pure SBR (with dot) corresponds to the results reported in ref (13); correlation length ξ (triangles; hSBR/dPS samples (up) and dSBR/hPS (down)) and average displacement at about 80 ps  (crosses: SBR; pluses: PS). The range of the relevant length scale for the α-process, 2*R_c_*, is marked with the gray area. The arrow marks the Kuhn length *l*_K_ of polymeric PS and the red area the range of *l*_K_ reported for SBR. The blue region shows the estimate of the end-to-end distance of the PS500 oligomers. Data correspond to *T* ≈ 300 K.

We can now compare the magnitudes of the different length scales identified in these samples that are relevant for diverse structural, dynamical and thermodynamical aspects, compiled in Figure 12. With regard to the structural aspects, we consider first the results provided by our X-ray experiments, which are described in detail in the Supporting Information. The average intermolecular distances obtained from the main peak position are in all cases *d*_chain_ ≈ 5 Å, in the range reported for other glass-forming polymers like 1,4-PB.^61^ Moreover, our study confirms also in this small PS oligomer and these blends the nanodomain picture previously reported for other systems with bulky side groups: inherent to the presence of these groups^62^ (phenyl rings, in this case), structural heterogeneities arise due to nanosegregation of main-chain and side-group atoms giving rise to a nanodomain peak. In the present PS oligomers, the nanodomain peak is less pronounced than in higher molecular weight samples, but even for so short PS chains nanosegregation is clear (see Figure S2.1). The results are similar to those found for the higher-molecular-weight (900 g/mol) oligomers. For the present neat SBR samples this peak is not resolvable (see Figure S2.1), but in a previous work^13^ we reported the existence of a weak peak for SBR in the *Q* range around 0.3 Å^–1^. This peak seems to be resolvable only beyond a threshold content of styrene units in the copolymer. This is about 20 wt % styrene, which was the case of the SBR in ref (13); here we have only 13.8 and 18%. Nanodomains persist in the blends (see Figure S2.1). In the mixtures, the peak would arise from the presence of styrene phenyls from both PS and SBR. Here we find in fact that in the blend with 80% SBR content, having thus a global content of styrene of about 30–34%, the peak is already visible (Figure S2.1). The inter-nanodomain distance *D* defined from the position of the nanodomain peak (see the Supporting Information) is represented in Figure 12. It increases with SBR content, having more linear chain portions. For intermediate to high PS concentration, this distance remains around 1 nm and can reach up to about 2 nm for pure SBR with high enough styrene content.^13^ This is the approximate value of the Kuhn length (*l*_K_ = 17 Å) in PS.^21,63^ The value of *l*_K_ in SBR is expected to depend on the microstructure and is not known for the present samples; from the literature we could also expect values in the 1–2 nm range (*l*_K_ = 12 Å, from ref (63); *l*_K_ = 16 Å, from ref (13)). Another structural parameter to be considered is the size of the structural units involved. For the oligomers, the end-to-end vector distance  is expected to be smaller than 2 nm, which is the value estimated from the 900 g/mol PS in ref (13). For the SBR chains involved in the present samples,  would be of the order of 23 nm for hSBR and 17 nm for dSBR, considering the reported value of 0.7 for the ratio , where *M* is the molecular mass.^64^ This means that this length is much larger than any other of the characteristic lengths represented in Figure 12.

We now move to the dynamic aspects. The spatial information provided by SANS allows, as explained above, to estimate the width of the distribution of TCF as a function of the explored volume through eq 4. We recall that for the sake of simplicity the volume considered is a sphere of radius *R*_c_. We call the such estimated width σ^SANS^(*R*_c_). This function depends on concentration. On the other hand, from the DSC analysis, we deduce concentration-dependent values of σ (we call them σ^DSC^), which account for the observed loss of equilibrium of the α-process. Thus, comparing the values obtained from both techniques (see Figure S4.8) and imposing σ^SANS^(*R*_c_) ≈ σ^DSC^, we can estimate what is the relevant volume “seen” in the DSC experiments. In this way, we find 2*R*_c_ ≈ 30 Å for this relevant length scale for the glass transition (see Figure 6). This value is in the same range found in our previous works on blends of PS oligomers of 900 g/mol and SBR of different microstructure (2*R*_c_ ≈ 25 Å,^5^ 2*R*_c_ ≈ 20 Å^13^). Note that in those cases the value of 2*R*_c_ was deduced from the study of the dielectric response, i.e., corresponding to the dynamics of the α-relaxation in equilibrium. The similarity of the 2*R*_c_ value with the Kuhn length of the polymers was brought forward in those works, as suggested in ref (65). However, the present results rule out *l*_K_ to be behind this length scale. The oligomers in our blends are smaller than a Kuhn segment (containing seven monomers), and the 2*R*_c_ value is similar to or even larger than those found in the previous cases. Thus, the size 2*R*_c_ cannot be related to any particular length scale associated with the chain size or conformation. On the other hand, 2*R*_c_ exceeds by a factor of about 20 the relevant length scale  characteristic for the “microscopic” glass transition, as observed from the EFWS. This may be defined as . As can be inferred from the above discussion, this characteristic length remains of the same order (around 1 Å) independently of choosing *T*_g_ or *T*_g,eff_ for its definition. We note that the atomic displacements at such relatively short times around the glass transition are characteristic for the local motions within the cage imposed by the neighboring chains, while characteristic times observed close to *T*_g_ for the α-process by relaxation methods such as dielectric spectroscopy are of the order of 1 s. In our case, they are τ_BDS_(*T*_g_) = 2.3 s (hSBR), 100 s (hPS), 2.9 s (dSBR), and 41 s (dPS) (see the Supporting Information). In fact, the characteristic time at the calorimetric *T*_g_ is the magnitude invoked in the proposed model^5^ to connect the component segmental dynamics in the blend above *T*_g_ with the way the equilibrium is lost when cooling toward the glassy state. We note that 2*R*_c_ values in the nanometer scale have been reported for polymers and other glass-forming systems.^66^

Finally, we consider the relevant length scale for TCF—the correlation length ξ. It strongly depends on concentration (see Figures 2 and 12), but for SBR contents about or higher than 50% it remains below 1 nm in the whole *T* range investigated. For high SBR concentrations, the values of ξ even approach the intermolecular distance *d*_chain_. Thus, under these conditions, the correlation length ξ is smaller than the chains’ dimensions—or similar to the smallest one—implying that the chains are randomly mixed. Only for the highest PS concentrations approaching the glass transition can the correlation length exceed the dimensions of the oligomers, being thus the mixture locally inhomogeneous.^23,67^

## Conclusions

For the homopolymers, we have demonstrated that the EFWS reflecting atomic displacements at some tens of picoseconds are sensitive to the onset of liquid-like motions across the calorimetric glass transition, even if the relaxation times associated with this phenomenon are of the order of tens of seconds, corroborating thereby previous findings. These displacements are of the order of 1 Å at the glass transition, supporting a Lindemann-like criterion. The microscopic insight into atomic displacements of a selected component in the blend has allowed determining its “microscopic” effective glass transition in the mixture. The values obtained were compared with the macroscopic counterparts deduced from the DSC analysis with a model based on TCF and SC. For the fast SBR component, we always find a coincidence of microscopic and microscopic effective glass transition temperatures. For the slow PS component, the situation is more complex. For the sample rich in SBR, the microscopic glass transitions of both components are similar, indicating that at this microscopic level the mixture is dynamically homogeneous. This leads to a kind of paradoxical situation in the sense that PS looses its equilibrium (as deduced from DSC) at a higher temperature than that where it undergoes its microscopic glass transition. This implies that there is a temperature range where this component feels a “liquid-like” microscopic environment but behaves “solid-like” from a macroscopic point of view. On the contrary, for intermediate and high PS contents, macroscopic and microscopic effective glass transitions of PS coincide (being thus different from those of SBR), and the system is heterogeneous at the microscopic level. We note that for high PS concentrations the system is close to phase separation. Heterogeneities are one of the sources of the non-Gaussian effects deduced for the atomic displacements. We have also found that the Lindemann-like criterion might also be applied for blends, where a simple mixing rule for the effective atomic mean-squared displacements determines the condition for glass transition.

Our comparative study has also allowed determining the characteristic length scale of the α-relaxation to be about 30 Å. This is similar to the values previously found for blends involving different components with different sizes. Its relation with the Kuhn length can be ruled out from this work because the oligomers do not meet this size. Inherent to the presence of bulky side groups in the chains, structural heterogeneities at the nanoscale arise due to nanosegregation of main chains and side-group atoms. They persist even though the small size of the oligomers and the dilution of phenyl rings with blending but are not expected either to be relevant to determine the characteristic length scale of the α-relaxation, which is apparently universal for glass-forming systems.

In addition to the above findings of basic interest, from an applied point of view we have shown that the model proposed to describe the effect of blending on experimental observables like DSC, BDS, or mechanical spectroscopy also works for a system of industrial interest with different characteristics than those where the model was previously applied, in terms of PS size (here below the Kuhn segment), SBR microstructure, and dynamic asymmetry. In addition, with the EFWS we provide microscopic and independent support for the validity of this model that allows predicting materials’ properties and thus valuable information in the industrial field.

## Supporting Information Available

(i) Magnitudes measured by the scattering experiments; (ii) X-ray diffraction results: local structure; (iii) SANS results: determination of *T*_s_ and χ parameter; (iv) DSC results and their modeling (PDF)

## References

[ref1] Colmenero J.; Arbe A. Recent progress on polymer dynamics by neutron scattering: From simple polymers to complex materials. J. Polym. Sci., Part B: Polym. Phys. 2013, 51, 87–113. 10.1002/polb.23178.

[ref2] Colmenero J.; Arbe A. Segmental dynamics in miscible polymer blends: recent results and open questions. Soft Matter 2007, 3, 1474–1485. 10.1039/b710141d. 32900101

[ref3] Gambino T.; Alegría A.; Arbe A.; Colmenero J.; Malicki N.; Dronet S.; Schnell B.; Lohstroh W.; Nemkovski K. Applying polymer blend dynamics concepts to a simplified industrial system. A combined effort by dielectric spectroscopy and neutron scattering. Macromolecules 2018, 51, 6692–6706. 10.1021/acs.macromol.8b00881.

[ref4] Gambino T.; Alegría A.; Arbe A.; Colmenero J.; Malicki N.; Dronet S. Modeling the high frequency mechanical relaxation of simplified industrial polymer mixtures using dielectric relaxation results. Polymer 2020, 187, 122051 10.1016/j.polymer.2019.122051.

[ref5] Shafqat N.; Alegría A.; Arbe A.; Malicki N.; Dronet S.; Porcar L.; Colmenero J. Disentangling the calorimetric glass-transition trace in polymer/oligomer mixtures from the modeling of dielectric relaxation and the input of small-angle neutron scattering. Macromolecules 2022, 55, 7614–7625. 10.1021/acs.macromol.2c00609. 36118597 PMC9477097

[ref6] Ayyagari C.; Bedrov D.; Smith G. D. Structure of atactic polystyrene: A molecular dynamics simulation study. Macromolecules 2000, 33, 6194–6199. 10.1021/ma0003553.

[ref7] Fujara F.; Petry W. Local motion around *T*_*g*_ in a molecular glass as observed by incoherent neutron scattering. Europhys. Lett. 1987, 4, 921–927. 10.1209/0295-5075/4/8/011.

[ref8] Niss K.; Dalle-Ferrier C.; Frick B.; Russo D.; Dyre J.; Alba-Simionesco C. Connection between slow and fast dynamics of molecular liquids around the glass transition. Phys. Rev. E 2010, 82, 021508 10.1103/PhysRevE.82.021508. 20866819

[ref9] Frick B.; Richter D.; Petry W.; Buchenau U. Study of the glass transition order parameter in amorphous polybutadiene by incoherent neutron scattering. Z. Physik B - Condensed Matter 1988, 70, 73–79. 10.1007/BF01320541.

[ref10] Frick B.; Richter D. The microscopic basis of the glass transition in polymers from neutron scattering studies. Science 1995, 267, 1939–1945. 10.1126/science.267.5206.1939. 17770103

[ref11] Dyre J. C.; Olsen N. B.; Christensen T. Local elastic expansion model for viscous-flow activation energies of glass-forming molecular liquids. Phys. Rev. B 1996, 53, 2171–2174. 10.1103/PhysRevB.53.2171. 9983702

[ref12] Dyre J. C. Colloquium: The glass transition and elastic models of glass-forming liquids. Rev. Mod. Phys. 2006, 78, 953–972. 10.1103/RevModPhys.78.953.

[ref13] Gambino T.; Shafqat N.; Alegría A.; Malicki N.; Dronet S.; Radulescu A.; Nemkovski K.; Arbe A.; Colmenero J. Concentration fluctuations and nanosegregation in a simplified industrial blend with large dynamic asymmetry. Macromolecules 2020, 53, 7150–7160. 10.1021/acs.macromol.0c01376.

[ref14] Bouty A.; Petitjean L.; Chatard J.; Matmour R.; Degrandcourt C.; Schweins R.; Meneau F.; Kwasniewski P.; Boue F.; Couty M.; Jestin J. Interplay between polymer chain conformation and nanoparticle assembly in model industrial silica/rubber nanocomposites. Faraday Discuss. 2016, 186, 325–343. 10.1039/C5FD00130G. 26791776

[ref15] Shafqat N.; Arbe A.; Colmenero J.; Porcar L. Concentration fluctuations and broadening of the dynamical response in dynamically asymmetric mixtures of industrial interest; Institut Laue-Langevin (ILL): 2020; 10.5291/ILL-DATA.6-04-282.

[ref16] Shafqat N.; Arbe A.; Colmenero J.; Natali F. Microscopic insight on component dynamics in polymeric mixtures of industrial interest; Institut Laue-Langevin (ILL): 2021; 10.5291/ILL-DATA.6-04-286.

[ref17] Shafqat N.; Arbe A.; Colmenero J.; Malicki N.; Dronet S.; Natali F. Isolating the glass transition of components in blends of polymers of industrial interest; Institut Laue-Langevin (ILL): 2020; 10.5291/ILL-DATA.CRG-2793.

[ref18] Natali F.; Peters J.; Russo D.; Barbieri S.; Chiapponi C.; Cupane A.; Deriu A.; Di Bari M. T.; Farhi E.; Gerelli Y.; Mariani P.; Paciaroni A.; Rivasseau C.; Schiro G.; Sonvico F. IN13 Backscattering Spectrometer at ILL: Looking for Motions in Biological Macromolecules and Organisms. Neutron News 2008, 19, 14–18. 10.1080/10448630802474083.

[ref19] Shafqat N.; Arbe A.; Colmenero J.; Malicki N.; Dronet S.; Mangin-Thro L. Component dynamics in polymeric mixtures of industrial interest; Institut Laue-Langevin (ILL): 2020; 10.5291/ILL-DATA.EASY-714.

[ref20] deGennes P. G. Scaling Concepts in Polymer Physics; Cornell University Press: Ithaca, NY, 1979.

[ref21] Rubinstein M.; Colby R. H. Polymer Physics; Oxford University Press: Oxford, 2003.

[ref22] Higgins J. S.; Benoit H. C. Polymers and Neutron Scattering; Oxford University Press: Oxford, 1997.

[ref23] Wignall G. D.; Melnichenko Y. B. Recent applications of small-angle neutron scattering in strongly interacting soft condensed matter. Rep. Prog. Phys. 2005, 68, 1761–1810. 10.1088/0034-4885/68/8/R02.

[ref24] Mortensen K. Characterization of Polymer Blends; Wiley-VCH Verlag GmbH & Co. KGaA: 2014; pp 237–268.

[ref25] Schwahn D.; Yee-Madeira H. Spinodal decomposition of the polymer blend deuterous polystyrene (d-PS) and polyvinylmethylether (PVME) studied with high resolution neutron small angle scattering. Colloid & Polymer Science 1987, 265, 867–875. 10.1007/BF01421814.

[ref26] Koizumi S. UCST behavior observed for a binary polymer mixture of polystyrene/poly(vinyl methyl ether) (PS/PVME) with a PS rich asymmetric composition as a result of dynamic asymmetry & imbalanced local stress, Viscoelastic Phase Separation, and Pinning by Vitrification. Soft Matter 2011, 7, 3984–3992. 10.1039/c0sm01215g.

[ref27] Schwahn D.; Pipich V.; Richter D. Composition and long-range density fluctuations in PEO/PMMA polymer blends: A result of asymmetric component mobility. Macromolecules 2012, 45, 2035–2049. 10.1021/ma2019123.

[ref28] Zetsche A.; Fischer E. Dielectric studies of the α-relaxation in miscible polymer blends and its relation to concentration fluctuations. Acta Polym. 1994, 45, 168–175. 10.1002/actp.1994.010450306.

[ref29] Katana G.; Fischer E. W.; Hack T.; Abetz V.; Kremer F. Influence of concentration fluctuations on the dielectric α-relaxation in homogeneous polymer mixtures. Macromolecules 1995, 28, 2714–2722. 10.1021/ma00112a017.

[ref30] Shenogin S.; Kant R.; Colby R. H.; Kumar S. K. Dynamics of miscible polymer blends: Predicting the dielectric response. Macromolecules 2007, 40, 5767–5775. 10.1021/ma070503q.

[ref31] Zorn R. On the evaluation of neutron scattering elastic scan data. Nuclear Instruments and Methods in Physics Research Section A: Accelerators, Spectrometers, Detectors and Associated Equipment 2009, 603, 439–445. 10.1016/j.nima.2009.02.040.

[ref32] Benedetto A.; Kearley G. Dynamics from elastic neutron scattering via direct measurement of the running time-integral of the van Hove distribution function. Sci. Rep. 2019, 9, 11284 10.1038/s41598-019-46835-z. 31375739 PMC6677729

[ref33] Benedetto A.; Kearley G. A quantitative comparison of the counting significance of van Hove integral spectroscopy and quasielastic neutron scattering. Sci. Rep. 2020, 10, 6350 10.1038/s41598-020-63193-3. 32286403 PMC7156666

[ref34] Benedetto A.; Kearley G. Experimental demonstration of the novel “van-Hove integral method (vHI)” for measuring diffusive dynamics by elastic neutron scattering. Sci. Rep. 2021, 11, 14093 10.1038/s41598-021-93463-7. 34238981 PMC8266890

[ref35] Doster W.; Diehl M.; Petry W.; Ferrand M. Elastic resolution spectroscopy: a method to study molecular motions in small biological samples. Physica B: Condensed Matter 2001, 301, 65–68. 10.1016/S0921-4526(01)00513-0.

[ref36] Doster W.; Diehl M.; Gebhardt R.; Lechner R.; Pieper J. TOF-elastic resolution spectroscopy: time domain analysis of weakly scattering (biological) samples. Chemical physics 2003, 292, 487–494. 10.1016/S0301-0104(03)00156-3.

[ref37] Doster W.; Nakagawa H.; Appavou M. S. Scaling analysis of bio-molecular dynamics derived from elastic incoherent neutron scattering experiments. J. Chem. Phys. 2013, 139, 045105 10.1063/1.4816513. 23902030

[ref38] Zorn R. Deviation from Gaussian behavior in the self-correlation function of the proton motion in polybutadiene. Phys. Rev. B 1997, 55, 6249–6259. 10.1103/PhysRevB.55.6249.

[ref39] Doster W.; Settles M. Protein-water displacement distributions. Biochim. Biophys. Acta 2005, 1749, 173–186. 10.1016/j.bbapap.2005.03.010. 15893505

[ref40] Doster W.; Busch S.; Gaspar A. M.; Appavou M.-S.; Wuttke J.; Scheer H. Dynamical transition of protein-hydration water. Phys. Rev. Lett. 2010, 104, 098101 10.1103/PhysRevLett.104.098101. 20367013

[ref41] Arbe A.; Genix A.-C.; Arrese-Igor S.; Colmenero J.; Richter D. Dynamics in poly(n-alkyl methacrylates): A neutron scattering, calorimetric, and dielectric study. Macromolecules 2010, 43, 3107–3119. 10.1021/ma902833h.

[ref42] Buchenau U.; Zorn R. A relation between fast and slow motions in glassy and liquid Selenium. Europhys. Lett. 1992, 18, 523–528. 10.1209/0295-5075/18/6/009.

[ref43] Stillinger F. H. A topographic view of supercooled liquids and glass formation. Science 1995, 267, 1935–1939. 10.1126/science.267.5206.1935. 17770102

[ref44] Zhou Y.; Vitkup D.; Karplus M. Native proteins are surface-molten solids: application of the Lindemann criterion for the solid versus liquid state. J. Mol. Biol. 1999, 285, 1371–1375. 10.1006/jmbi.1998.2374. 9917381

[ref45] Katava M.; Stirnemann G.; Zanatta M.; Capaccioli S.; Pachetti M.; Ngai K. L.; Sterpone F.; Paciaroni A. Critical structural fluctuations of proteins upon thermal unfolding challenge the Lindemann criterion. Proc. Natl. Acad. Sci. U.S.A. 2017, 114, 9361–9366. 10.1073/pnas.1707357114. 28808004 PMC5584445

[ref46] Xia X.; Wolynes P. G. Fragilities of liquids predicted from the random first order transition theory of glasses. Proc. Natl. Acad. Sci. U. S. A. 2000, 97, 2990–2994. 10.1073/pnas.97.7.2990. 10737779 PMC16179

[ref47] Novikov V. N.; Sokolov A. P. Universality of the dynamic crossover in glass-forming liquids: A “magic” relaxation time. Phys. Rev. E 2003, 67, 031507 10.1103/PhysRevE.67.031507. 12689073

[ref48] Larini L.; Ottochian A.; De Michele C.; Leporini D. Universal scaling between structural relaxation and vibrational dynamics in glass-forming liquids and polymers. Nat. Phys. 2008, 4, 42–45. 10.1038/nphys788.

[ref49] Shafqat N.; Alegría N.; Malicki A.; Dronet S.; Mangin-Thro L.; Frick B.; Colmenero J.; Arbe A. Microscopic versus macroscopic glass transition(s) in blends of industrial interest. EPJ. Web Conf 2022, 272, 01008 10.1051/epjconf/202227201008. PMC10019463 36938513

[ref50] Dalle-Ferrier C.; Simon S.; Zheng W.; Badrinarayanan P.; Fennell T.; Frick B.; Zanotti J. M.; Alba-Simionesco C. Consequence of excess configurational entropy on fragility: The case of a polymer-oligomer blend. Phys. Rev. Lett. 2009, 103, 185702 10.1103/PhysRevLett.103.185702. 19905814

[ref51] Narros A.; Arbe A.; Alvarez F.; Colmenero J.; Richter D. Atomic motions in the *αβ*-merging region of 1,4-polybutadiene: A molecular dynamics simulation study. J. Chem. Phys. 2008, 128, 224905 10.1063/1.2937733. 18554051

[ref52] Narros A.; Alvarez F.; Arbe A.; Colmenero J.; Richter D.; Farago B. Hydrogen motions in the alpha-relaxation regime of poly(vinyl ethylene): A molecular dynamics simulation and neutron scattering study. J. Chem. Phys. 2004, 121, 3282–3294. 10.1063/1.1772761. 15291640

[ref53] Colmenero J.; Alvarez F.; Arbe A. Self-motion and the α relaxation in a simulated glass-forming polymer: Crossover from Gaussian to non-Gaussian dynamic behavior. Phys. Rev. E 2002, 65, 041804 10.1103/PhysRevE.65.041804. 12005863

[ref54] Kob W.; Andersen H. C. Testing mode-coupling theory for a supercooled binary Lennard-Jones mixture I: The van Hove correlation function. Phys. Rev. E 1995, 51, 4626–4641. 10.1103/PhysRevE.51.4626. 9963176

[ref55] Kob W.; Donati C.; Plimpton S. J.; Poole P. H.; Glotzer S. C. Dynamical heterogeneities in a supercooled Lennard-Jones liquid. Phys. Rev. Lett. 1997, 79, 2827–2830. 10.1103/PhysRevLett.79.2827.

[ref56] Caprion D.; Matsui J.; Schober H. R. Dynamic heterogeneity of relaxations in glasses and liquids. Phys. Rev. Lett. 2000, 85, 4293–4296. 10.1103/PhysRevLett.85.4293. 11060621

[ref57] Hurley M. M.; Harrowell P. Non-Gaussian behavior and the dynamical complexity of particle motion in a dense two-dimensional liquid. J. Chem. Phys. 1996, 105, 10521–10526. 10.1063/1.472941.

[ref58] Kämmerer S.; Kob W.; Schilling R. Test of mode coupling theory for a supercooled liquid of diatomic molecules. I. Translational degrees of freedom. Phys. Rev. E 1998, 58, 2131–2140. 10.1103/PhysRevE.58.2131.

[ref59] Caprion D.; Schober H. R. Structure and relaxation in liquid and amorphous selenium. Phys. Rev. B 2000, 62, 3709–3716. 10.1103/PhysRevB.62.3709.

[ref60] Arbe A.; Colmenero J.; Alvarez F.; Monkenbusch M.; Richter D.; Farago B.; Frick B. Experimental evidence by neutron scattering of a crossover from Gaussian to non-Gaussian behavior in the α relaxation of polyisoprene. Phys. Rev. E 2003, 67, 51802 10.1103/PhysRevE.67.051802. 12786170

[ref61] Frick B.; Richter D.; Ritter C. Structural changes near the glass transition–neutron diffraction on a simple polymer. EPL (Europhysics Letters) 1989, 9, 557–562. 10.1209/0295-5075/9/6/011.

[ref62] Moreno A. J.; Arbe A.; Colmenero J. Structure and dynamics of self-assembled comb copolymers: Comparison between simulations of a generic model and neutron scattering experiments. Macromolecules 2011, 44, 1695–1706. 10.1021/ma102545n.

[ref63] Fetters L. J.; Lohse D. J.; Colby R. H. In Physical Properties of Polymers Handbook; Mark J. E., Ed.; Springer: New York, 2007; pp 447–454.

[ref64] Fetters L. J.; Lohse D. J.; Milner S. T.; Graessley W. W. Packing length influence in linear polymer melts on the entanglement, critical, and reptation molecular weights. Macromolecules 1999, 32, 6847–6851. 10.1021/ma990620o.

[ref65] Lodge T. P.; McLeish T. C. B. Self-concentrations and effective glass transition temperatures in polymer blends. Macromolecules 2000, 33, 5278–5284. 10.1021/ma9921706.

[ref66] Berthier L.; Biroli G.; Bouchaud J.-P.; Cipelletti L.; Masri D. E.; L’Hôte D.; Ladieu F.; Pierno M. Direct experimental evidence of a growing length scale accompanying the glass transition. Science 2005, 310, 1797–1800. 10.1126/science.1120714. 16357256

[ref67] Yun S. I.; Melnichenko Y. B.; Wignall G. D. Small-angle neutron scattering from symmetric blends of poly(dimethylsiloxane) and poly(ethylmethylsiloxane). Polymer 2004, 45, 7969–7977. 10.1016/j.polymer.2004.09.050.

