# Supplementary material for: Microscopic versus Macroscopic Glass Transitions and Relevant Length Scales in Mixtures of Industrial Interest
Source: Macromolecules. 2023 Feb 28;56(5):2149–63. doi: 10.1021/acs.macromol.2c02368 (PMC10019463; doi:10.1021/acs.macromol.2c02368)
Supplement: Supplementary file 1 — ma2c02368_si_001.pdf [file ma2c02368_si_001.pdf]

Supporting Information:
Microscopic versus Macroscopic Glass Transitions and Relevant Length Scales in Mixtures of Industrial Interest

Numera Shafqat,[†,‡] Angel Alegría,[†,⁀] Nicolas Malicki,[‡] Séverin Dronet,[‡] Francesca Natali,[⟦] Lucile Mangin-Thro,[&] Lionel Porcar,[&] Arantxa Arbe,[*,†] and Juan Colmenero[*,†,⁀,⊥]

†*Centro de Física de Materiales (CSIC, UPV/EHU) and Materials Physics Center MPC, Paseo Manuel de Lardizabal 5, E-20018 San Sebastián, Spain*

‡*Manufacture Française des Pneumatiques MICHELIN, Site de Ladoux, 23 place des Carmes Déchaux, F-63040, Clermont-Ferrand, Cedex 9, France*

⁀*Departamento de Polímeros y Materiales Avanzados: Física, Química y Tecnología (UPV/EHU), Facultad de Química, E-20018 San Sebastián, Spain*

⟦ *CNR-IOM, OGG, 71 avenue des Martyrs, 38043, Grenoble, Cedex 9, France*

& *Institut Laue-Langevin, 71 avenue des Martyrs, Grenoble Cedex 9, 38042, France*

⊥*Donostia International Physics Center (DIPC), Paseo Manuel de Lardizabal 4, E-20018 San Sebastián, Spain*

*E-mail: a.arbe@csic.es; juan.colmenero@ehu.eus

1. MAGNITUDES MEASURED BY THE SCATTERING EXPERIMENTS

As a representative example, Figure S1.1 shows the differential scattering cross section measured by SANS on the sample with 50/50 composition (50h). These data are combined with the information obtained from D7. Using both instruments, the Q-range from about 0.003 to 2.5Å^{-1} has been covered. This is equivalent to spatial scales (~1/Q) ranging from about the bond length to several nanometers. In addition to the diffraction experiments, we have also carried out EFWS on IN13 which cover the high-Q regime $0.52 \leq Q \leq 4.5$ Å^{-1}. We address in the following the origin of the contributions to the scattered intensity, taking Figure S1.1 as illustration.

The interaction of a given nucleus with neutrons is characterized by the scattering length b. This magnitude depends on the relative orientation of the neutron-nuclear spin pairs and varies from one isotope to another. The huge difference of the scattering length values for hydrogen and deuterium (b_H = -3.74 fm, b_D = 6.67 fm; $b_D \sim b_C$ = 6.65 fm) produces a high contrast between h/d isotopically labeled macromolecules. Therefore, SANS diffraction experiments accessing low values of Q —exploring thus large-scale properties— on mixtures where one of the components is protonated and the other is deuterated are highly sensitive to thermally driven concentration fluctuations (TCF). In such a Q-range also long-range density fluctuations contribute to the scattered intensity, though this contribution is usually expected to be much less important than that of TCF. Both, concentration and density fluctuations, give rise to coherent scattering. Toward high-Q values —local length scales— the contribution of TCF tends to vanish, and coherently scattered neutrons reflect instead the (partial) structure factors revealing the

short-range order in the sample. Its main manifestation in glass-forming systems like polymers is the broad peak usually appearing in the Q-range of about 1 Å^{-1} reflecting inter-molecular correlations.

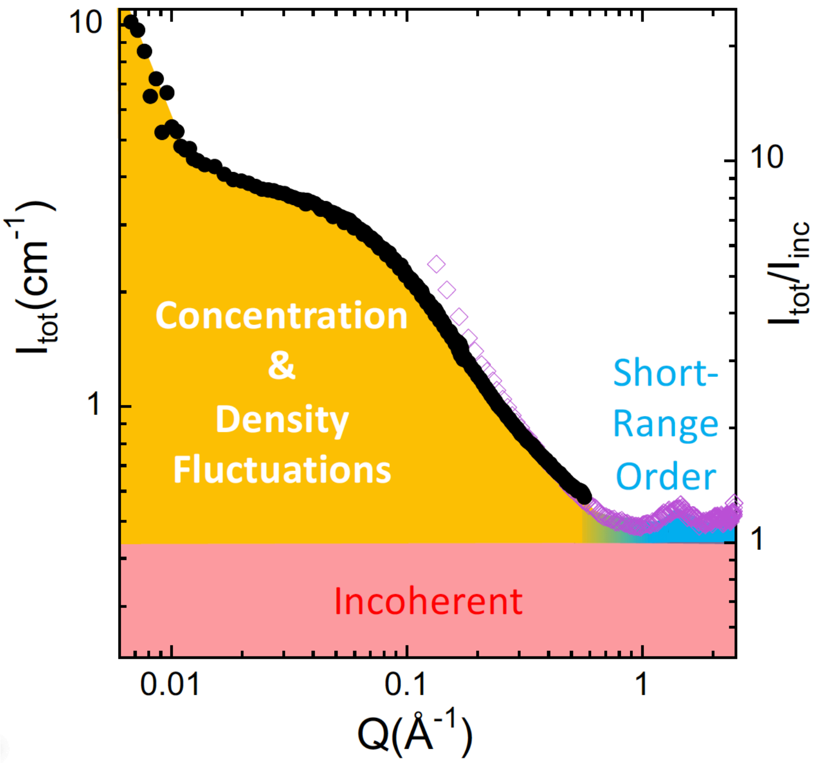

Figure S1.1. SANS (circles, scale on the left) and D7 (diamonds, scale on the right) results on the 50h-sample (50% hSBR / 50% dPS composition) around RT. Areas with different colors show the different contributions to the differential scattering cross-section: the Q-independent incoherent scattering (red) and the coherent contributions mainly dominated by concentration and long-range density fluctuations at low Q (yellow) and reflecting the short-range order at high Q (blue).

Superimposed to these coherent contributions incoherent scattering of very different nature is also present in the measured signal. The incoherent differential scattering cross section is Q-independent and appears as a flat background in diffraction experiments. Incoherent scattering is particularly important by hydrogen nuclei. The incoherent scattering cross-section of H amounts to $\sigma_{inc}^{H} \approx 80$ barn, while its coherent cross-section is $\sigma_{inc}^{HD} \approx 2$ barn. In general, in hydrogenated samples, or samples containing hydrogens,

the incoherent cross section (summed up over all nuclei of the system) is much higher than the coherent one (see Table 3 in the main text). However, this does not imply that in a given Q-region the incoherent scattering always dominates the spectrum, since —as we can see in the example of Fig. S1.1— coherent scattering strongly depends on Q. The neutron spin is flipped with 2/3 probability in incoherent scattering due to nuclear spin disorder, whereas no flip occurs in the case of coherent scattering[1]. Thanks to this property, polarization analysis of the scattered intensities allows distinguishing between these two kinds of phenomena. This is the principle applied in the diffraction experiments carried out by means of D7. As can be seen in Fig. S1.1, D7 results tell us that for the sample with 50% hSBR / 50% dPS composition the incoherent contribution dominates the scattered intensity in the high-Q range above ≈ 0.5 Å^{-1}. This is the range explored by IN13.

On IN13, no polarization analysis is performed. Therefore, the intensity recorded in the EFWS has both, incoherent and coherent contributions. From the D7 results also for other blends shown in Fig. 3 we can infer that in the IN13 Q-window the coherent contribution from TCF is much smaller than the incoherent one, and that the scattered signal is dominated by the incoherent contribution. The exception is the sample rich in the deuterated component, but only in the neighborhood of the structure factor peak (around 1.3 Å^{-1}). D7 experiments were restricted to the 80 and 50 % SBR compositions. For the 20 % SBR samples, we could expect qualitatively similar results as those obtained for the 80 % SBR systems with opposite labeling.

Even if deuterons also scatter incoherently, their incoherent cross-section (2barn) is negligible compared to that of hydrogen. Carbon scatters only coherently. Thus, incoherent scattering from the samples investigated basically stems from hydrogens.

Contrarily to coherent scattering, incoherent scattering does not tell anything about structural features of the sample. However, it carries very valuable information on the self-atomic displacements that can be unveiled by energy-transfer ($\hbar\omega$) resolved experiments. The incoherent double differential scattering cross-section is in fact proportional to the incoherent scattering function $S_{inc}(Q, \omega)$. $S_{inc}(Q, \omega)$ is the Fourier transform of the intermediate incoherent scattering function $I_{inc}(Q, t)$, and the double Fourier transform yields the self-part of the van Hove correlation function $G_s(r, t)$. In the classical limit, $G_s(r, t)$ is the probability of a given nucleus to be at a distance *r* from the position where it was located at a time *t* before. Incoherent scattering looks at correlations between the positions of the same nucleus at different times. IN13 EFWS record the intensity of neutrons scattered with energy tranfers smaller or equal to the instrument energy resolution ($\delta\hbar\omega \approx 8$ μeV). Though not strictly exact,[2,3] the information extracted from the EFWS can be considered as an approximation to the intermediate incoherent scattering function at the time corresponding to the IN13 resolution $I_{inc}(Q, t_R)$ (see, e.g. [4]), with $t_R = \hbar / \delta\hbar\omega \approx 80$ ps.

Moving to X-Rays, the weights of the contributing correlations to the structure factor measured by diffraction are the Q-dependent atomic scattering factors for X-Rays. Since this probe mainly interacts with the electrons, these weights are proportional to the atomic number and no sensitive to isotopic labeling. Therefore, due to lack of contrast, the low-Q scattering from TCF, so prominent in the SANS experiments on our samples, is absent in the X-Ray diffraction measurements (see Fig. S1.2 as an example).

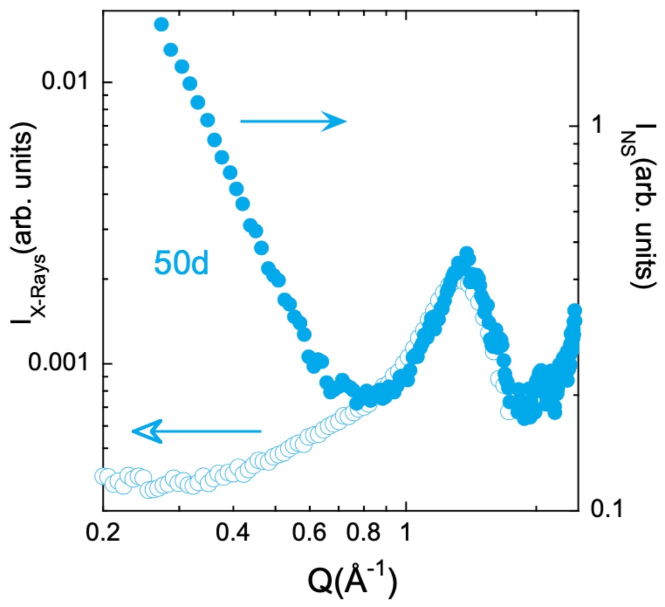

Figure S1.2. Comparison between D7 (filled symbols) and X-Ray (empty symbols) results for the coherent scattering of the 50d sample.

2. X-RAY DIFFRACTION RESULTS: LOCAL STRUCTURE

Since the X-Ray diffraction results are free from low-Q contributions from TCF, they were used to study the short-range order and possible nano-domain structuration of phenyl rings in the samples. Figure S2.1 shows the results obtained for the different blends and homopolymers. Panel (a) corresponds to samples based on hSBR and dPS and panel (b), on dSBR and hPS. Data have been normalized to their maximum value. This maximum is located at around $Q_{max} \approx 1.3$ Å^{-1}, and corresponds to correlations between pairs of atoms belonging to nearest neighboring chains. From its position, using the Bragg approximation, we can infer the average inter-chain distance $d_{chain}=2\pi/Q_{max}$. This is very close and nearly indistinguishable for all the samples: about 4.7 Å for SBR ($Q_{max} \approx 1.33$ Å^{-1}) and 4.8 Å for PS ($Q_{max} \approx 1.30$ Å^{-1}), and in between for the blends. In addition, we can clearly see a peak centered at about 0.68 Å^{-1} for the PS homopolymers. This peak has been assigned to phenyl ring - phenyl ring correlations and thereby attributed to the nano-

segregation of phenyl rings and main-chain atoms in PS[5,6]. Our experiments thus prove that this kind of nano-seggregation persists in oligomers as small as those here investigated. The location of this peak would be determined by the inter-domain distance *D*. On the other hand, the pure SBR samples do not show any clear hint for the presence of such a low-*Q* peak. In the blends there is an extra-intensity with respect to that corresponding to the pure SBR results in the low-*Q* region, which increases with increasing PS content. This feature could be attributed to the persistence of nano-seggregation of the phenyl rings with respect to the main-chains also in the blends. To analyze this contribution, we have assumed in a first approximation that the scattered intensity is the simple addition of a low-*Q* peak arising from the nano-domain structure and a high-*Q* peak reflecting the pure inter-chain correlation contributions. To represent the latter, we have used the pure SBR results. Thus, the difference between the total pattern and the SBR pattern would in a first order correspond to the nano-domain peak. This difference is shown in the insets of Fig. S2.1. Using a Gaussian function to describe it we have obtained the position of this peak. From the Bragg approximation, the inter-domain distance *D* has been deduced as function of composition. The values found are represented in Figure 12 together with those obtained for the average inter-molecular distances d_{chain}.

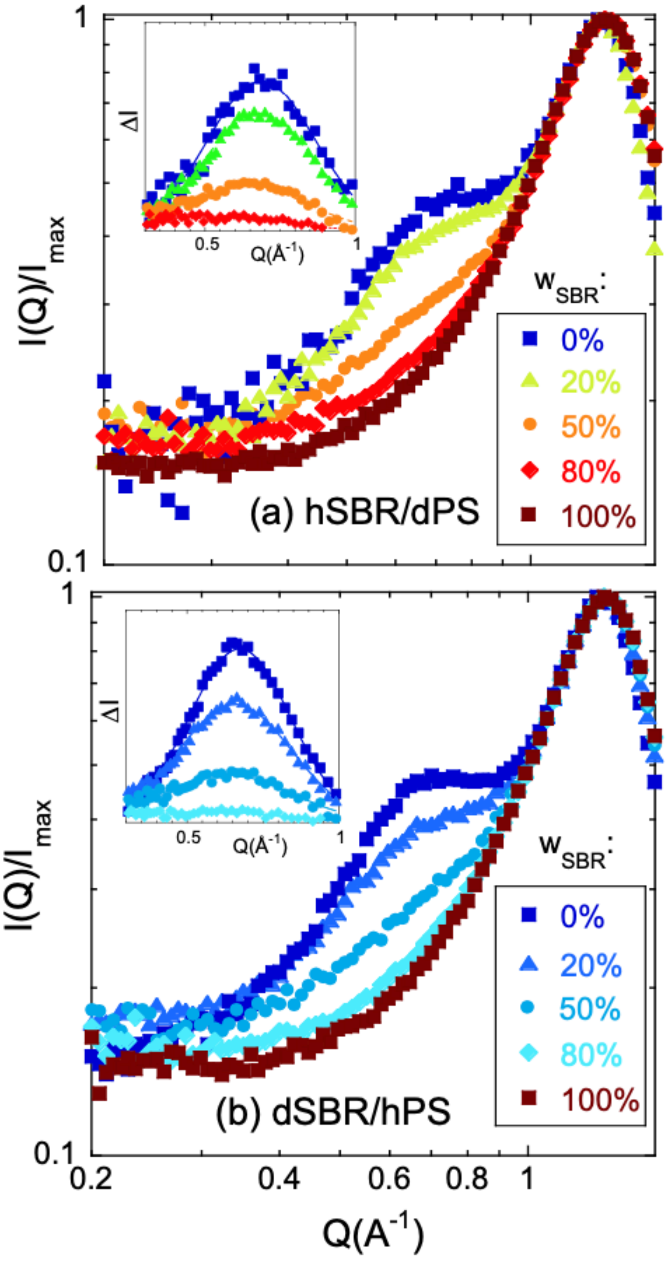

Figure S2.1. X-Ray diffraction results on the samples composed by (a) hSBR and dPS and (b) dSBR and hPS, at the SBR-concentrations indicated, normalized to their value at the main peak. The insets show the peak resulting of subtracting the pure SBR results from each of the patterns. Lines are fits of a Gaussian function.

3. SANS RESULTS: DETERMINATION OF T_s AND χ-PARAMETER

As explained in the manuscript, the values of the spinodal temperatures T_s were obtained by extrapolating the law $I_{OZ}^{-1}(0) \propto T^{-1}$ to low temperatures and deducing the value at which $I_{OZ}^{-1}(0)$=0. The construction is shown in Figure S3.1 for the samples with 20% and 50% SBR concentration considered in the manuscript and in addition for samples with 65% SBR content (not included in the manuscript because they were not investigated by IN13). The results on the 80% blends are out of the scale in this plot.

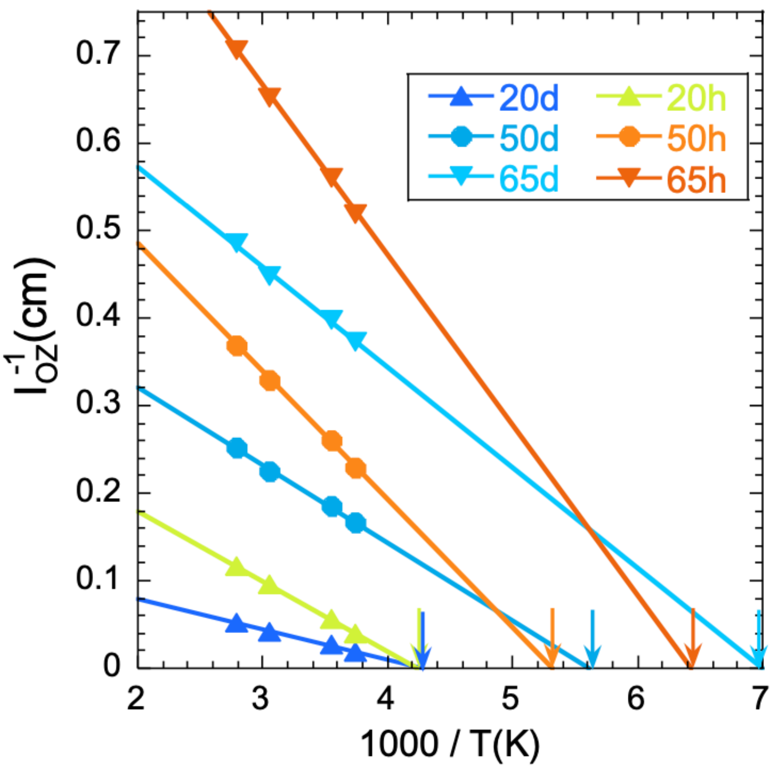

Figure S3.1. Inverse temperature dependence of the inverse of the OZ amplitudes corresponding to the samples with 20, 50 and 65% SBR concentration. Lines are linear regression fits and arrows mark the locations of the spinodal temperatures.

From the SANS results the interaction parameter between the two components χ was also obtained from the measured amplitudes of the OZ contribution, applying the RPA expression:

$$\frac{(\Delta\rho)^2}{I_{OZ}(0)} = \frac{1}{N_{SBR}\varphi_{SBR}v_{SBR}} + \frac{1}{N_{PS}(1-\varphi_{SBR})v_{PS}} - \frac{2\chi}{\sqrt{v_{SBR}v_{PS}}} \quad \text{(S3.1)}$$

where N_x is the number of monomers of species x and the rest of the parameters are defined in the manuscript (see Figure S3.2). The values obtained are shown in the insert of the figure. They follow the laws $\chi = 0.0274 + 16.36/T$ (hSBR/dPS) and $\chi = 0.0535 + 7.36/T$ (dSBR/hPS).

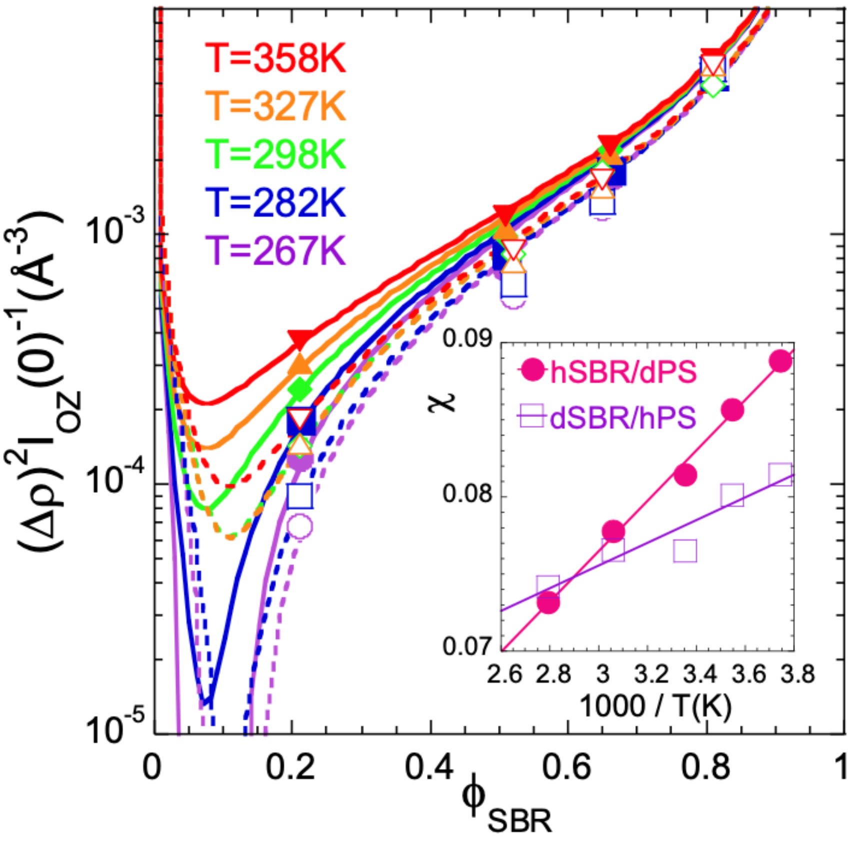

Figure S3.2. SBR volume fraction dependence of the inverse of S(0) at the temperatures indicated. Lines are fits of eq. S3.1. Solid symbols and lines correspond to the hSBR/dPS samples; empty symbols and dotted lines to the dSBR/hPS samples. The inset shows the inverse temperature dependence of the χ-parameter obtained from these fits, described by the laws indicated in the text.

4. DSC RESULTS AND THEIR MODELING

The approach proposed here to model the DSC behavior in the SBR/PS blends is based on a direct connection between DSC and broad band dielectric spectroscopy (BDS) experiments. We first analyze the glass transition and the dielectric relaxation of the neat components and afterward we model the calorimetric traces of the blends.

(i) Calorimetric Traces of the Glass Transition

Figure S4.1 shows an example of the results on the reversible part of the heat flow corresponding to the blend hSBR/dPS with w_{SBR}=0.50 composition. The glass transition manifests as a step in this function, and the value of T_g is commonly taken as the inflection point of reversible heat flow. To give account for the broadening of this transition, the construction illustrated in this figure for the case of the blend results is usually made. For this sample, we deduce a value of T_g = 230 K, with initial and final glass-transition temperature values of $T_{g,init}$ = 221 and $T_{g,fin}$ = 238 K respectively (see the arrows). The temperature-derivative of rev heat flow is also included in this figure; the glass transition is reflected as a peak in this function, where the position of the maximum corresponds to the inflection point of rev heat flow and thus directly gives the value of T_g as usually defined. This function also reflects very clearly the width of the glass transition process and may allow resolving multiple transitions, if present in the sample[7,8].

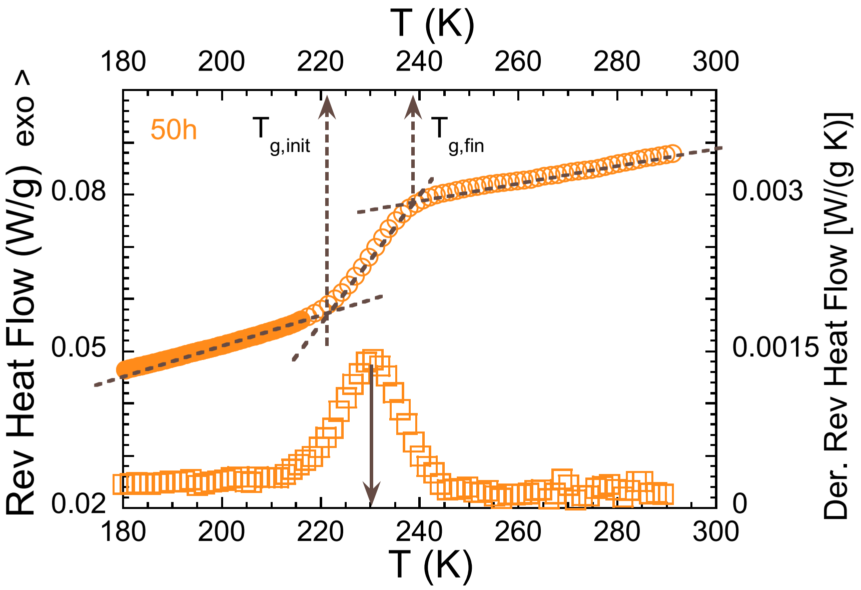

Figure S4.1. Reversible heat flow (circles) and its temperature derivative (squares) during cooling at 3 K/min for the blend 50h. The filled symbols correspond to the linear description of the glassy part. Dotted lines illustrate the usual construction to determine the initial and final glass-transition temperature values. Solid arrow marks the glass-transition temperature as directly determined from the maximum of the derivative.

In order to analyze the contribution to the experimental DSC trace of the segmental dynamics responsible for the glass transition, first the glassy behavior has been accounted for with a linear function (for the sake of simplicity) and subtracted from the DSC cooling scan of the reversible heat flow (Figure S4.1). We have used this procedure for the homopolymers as well for the blends. The resulting calorimetric traces for the hSBR/dPS and dSBR/hPS systems respectively, that will be used for the following analysis are shown in Figure S4.2 and will be referred to as segmental heat capacity, s-C_p. The behavior at temperatures well above T_g for all samples nearly superimposes and can be approximately described by a power law (T^{-n}) with n=2. It is worth mentioning that the subtraction of a linear function does not alter the location of the inflection point.

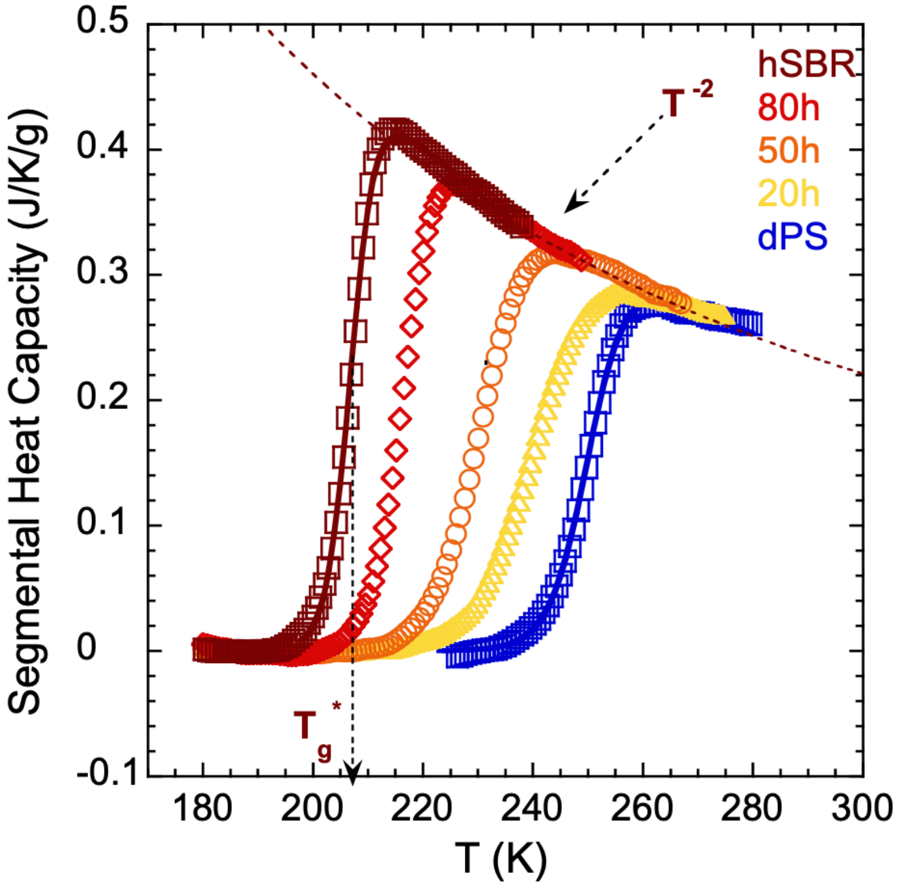

Figure S4.2. Calorimetric traces after the subtraction of the glassy part; same procedure has been applied on the neat components and the mixtures hSBR/dPS. The solid lines fitting the neat polymers data were obtained by using eq. S4.2 with parameters given in Table S1.

(ii) Dielectric Relaxation of Neat Components

Figure S4.3 shows an example of the dielectric loss as a function of the frequency for the two pure polymers, hSBR and dPS (panel on the left) – dSBR and hPS (panel on the right), at temperatures where the main peak is well-centered in the experimental frequency window. In this representation we have used $Tan\ \delta \equiv \frac{\varepsilon''}{\varepsilon'}$ to minimize the impact of sample geometry changes that could occur for measurements over a large temperature interval. The main loss peak is due to the segmental dynamics or α-relaxation.

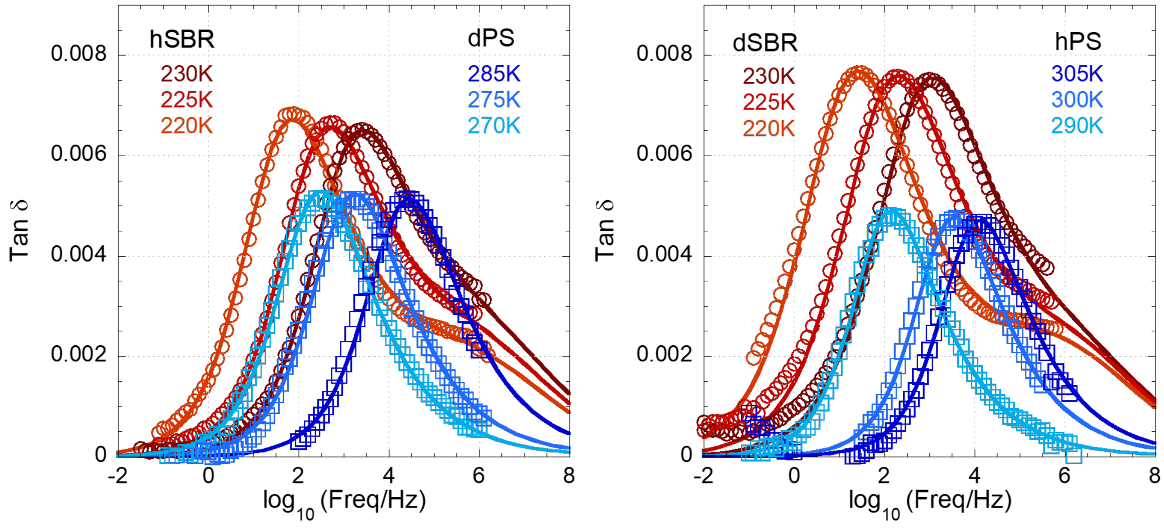

Figure S4.3. Frequency dependence of the dielectric tan δ for SBR (empty circles) and for PS (empty squares) at different temperatures. The solid lines represent the fits by means of the Havriliak-Negami equation for the α-relaxation and the addition of the β-relaxation calculated by extrapolating the lower temperature description, as explained in Ref. 11.

The dielectric α-relaxations can be described by means of the Havriliak-Negami (HN) equation;[9,10] and the necessary β-relaxation contribution has been taken in account by using a Gaussian function, following previous work[11].

Figure S4.3 shows that in this way a good description of the experimental data is obtained; the low-frequency increase of the data is due to conductivity effects, not considered in this analysis. The characteristic time at each temperature can be defined as the inverse of the angular frequency at the dielectric loss-permittivity maximum ($\tau_{max} \equiv \omega^{-1}{}_{max}$) of the α-relaxation process as calculated from the HN fitting parameters. Figure S4.4 shows the temperature dependence of the τ_{max} for SBR and PS. The lines in the figure correspond to the data description by means of Vogel-Fulcher-Tammann (VFT) equation: [12-14]

$$\tau(T) = \tau_\infty exp[DT_0/(T - T_0)] \qquad \text{(S4.1)}$$

In the fits we kept constant the prefactor value τ_∞=10^{-13}s in the VFT equation. Table S1 includes the values obtained for the fragility parameter, D, and the Vogel temperature T_0.

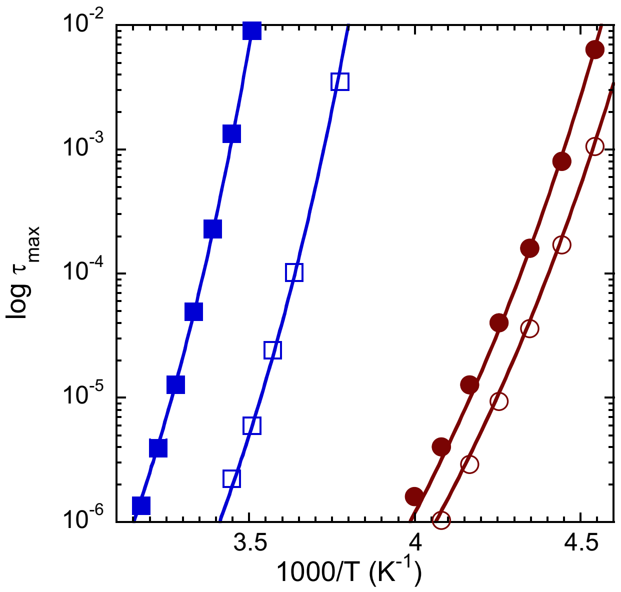

Figure S4.4. Temperature dependence of the characteristic times defined from the inverse of the frequencies of the dielectric loss maxima for the α-relaxation process of the neat samples investigated hSBR and dPS (empty symbols), dSBR and hPS (filled symbols). Circles correspond to SBR and squares to PS. The lines stand for the fits by means of the VFT equation.

Table S1: Parameters relevant for the description of the segmental dynamics time and for the calorimetric data.

sample	D	T_0/K	$\tau_{max}(T_g^*)/s$	δ/K	ΔC_{pg}/Jg^{-1}K^{-1}	T_g^*/K
hSBR	8.6	161.80	2.33	0.23	0.45	207.01
dPS	6.3	210.75	40.66	0.14	0.32	250.22
dSBR	8.6	163.58	2.91	0.21	0.39	208.96
hPS	6.3	228.00	99.83	0.19	0.23	269.59

(iii) Calorimetric Traces of the Neat Components

A simple full characterization of the homopolymers' DSC behavior has been done in order to establish the connection between the segmental dynamics time and the DSC data. Following previous work,[11] the description of the DSC traces in the glass transition range for the neat polymers required quantifying the three main quantities for each component: a characteristic temperature, a measure of the width of the glass transition range and the associated heat capacity jump. A simple but satisfactory way to describe the experimental segmental heat capacity of the neat polymers near their respective glass transitions is by combining a sigmoidal function with a T^{-2} law as:

$$\text{s-C}_p = \Delta C_{pg}\left(\frac{T_g^*}{T}\right)^2 \frac{1}{1+e^{(T_g^*-T)/\delta}} \tag{S4.2}$$

where ΔC_{pg} is the heat capacity jump, δ measures the width of the glass transition range and T_g^* is a characteristic temperature defined as the inflection point of the sigmoidal function. As can be appreciated in Figure S4.2, the description of the experimental data for the neat components, for example hSBR and dPS, is very good. The parameters determined by fitting the curves are given in Table S1.

Using the neat polymers DSC and BDS results we can connect the DSC T_g^* value and the segmental relaxation time evaluated at this temperature $\tau(T_g^*)$ for the two components. From the analysis of the pure polymers, respectively, we find that the relationship between the dielectric α-relaxation time and the calorimetric T_g^* is $\tau_g^{hSBR} \equiv \tau^{hSBR}(T_g^*) = 2.3\ s$, $\tau_g^{dPS} \equiv \tau^{dPS}(T_g^*) = 41\ s$, $\tau_g^{dSBR} \equiv \tau^{dSBR}(T_g^*) = 2.9\ s$ and $\tau_g^{hPS} \equiv \tau^{hPS}(T_g^*) = 100\ s$.

(iv) Composition Dependence of the Glass Transition of the Blends

The glass transition processes of the blends manifest broad features in the range between the $T_g s$ of the pure components, as can be observed in figure S4.2.

Figure S4.5 shows the composition dependence of the glass transition temperatures defined from the inflection point. As expected T_g decreases monotonously as we increase the content of SBR in the blends.

Following the scheme of a related work,[11] we have first described the whole set of data using the using the Gordon-Taylor (G-T) equation[15,16]

$$T_g^{blend} = \left[(1-\varphi)T_g^{PS} + k_{G-T}\varphi T_g^{SBR}\right] / \left[(1-\varphi) + k_{G-T}\varphi\right] \qquad \text{(S4.3)}$$

where φ is the weight fraction of SBR and a fitting parameter k_{G-T} is introduced.[17] The G-T equation includes the Fox equation[18]

$$1/T_g^{Blend} = \varphi/T_g^{SBR} + (1-\varphi)/T_g^{PS} \qquad \text{(S4.4)}$$

used in previous works as a particular case (when $k_{G-T} = T_g^{PS}/T_g^{SBR}$).

The G-T equation provides a satisfactory fit of the data for both systems (Figure S4.5) and yields to $k_{G-T} = 1.3$ for the hSBR/dPS and to $k_{G-T} = 1.6$ for the inverse labeling dSBR/hPS. The fit by the Fox equation is shown in Fig. S4.5 as dotted lines for comparison. While for the hSBR/dPS blends it works rather well, for the dSBR/hPS blends deviations are found that can well be accounted for by the Gordon-Taylor equation.

The physical meaning of the k_{G-T} parameter is well established for strictly athermal incompressible mixtures. In this case, k_{G-T} reflects the different contributions of each

component to the jump in heat capacity[19]: k_{G-T}= $\Delta C_p^{A}/\Delta C_p^{B}$ (A=SBR and B=PS in the present case). The Fox equation thus results as a particular case of the Gordon and Taylor equation that would work if: $\Delta C_p^{A}\ T_g^{A}=\Delta C_p^{B}\ T_g^{B}$. However, the Gordon and Taylor equation also usually works well for cases where there are weak interactions. Then, the value of k_{G-T} is also influenced by the strength of the interaction. For non-ideal (real) mixtures with varying degrees of intermolecular interactions, the k_{G-T} parameter is largely used for data fitting purposes only[20].

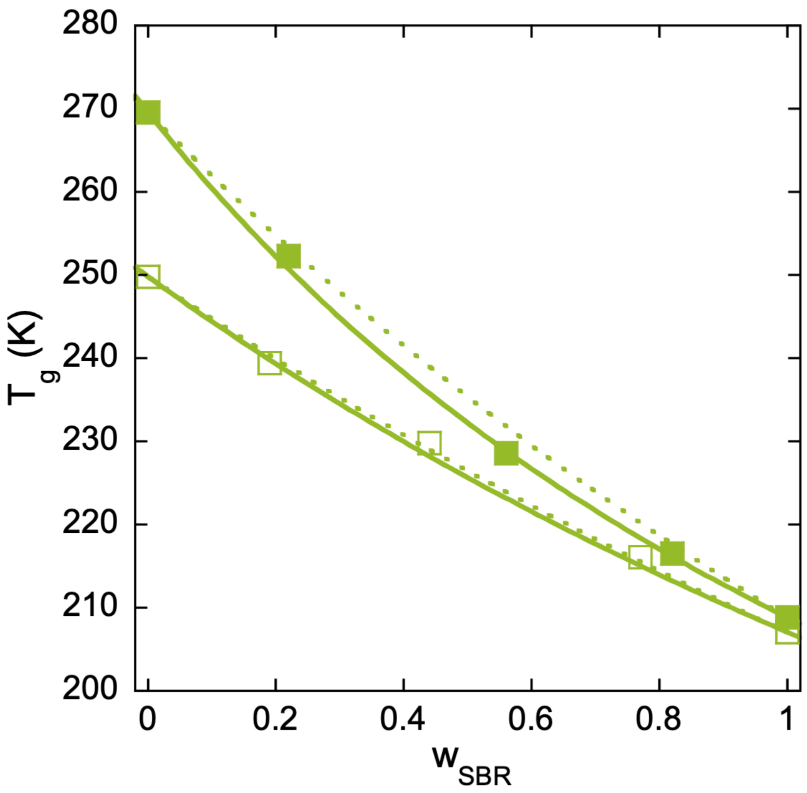

Figure S4.5. Evolution of the glass-transition temperature as a function of composition for hSBR/dPS blends (empty squares) and for dSBR/hPS (filled squares). The solid lines represent the description by the Gordon-Taylor equation (eq. S4.3) for the blends. Dotted lines are the Fox descriptions (eq. S4.4).

(v) Modeling the Calorimetric Traces of the Blends

The model developed during recent years of the segmental dynamics of miscible polymer blends[11,21,22] is based on two major ingredients: the thermally driven concentration fluctuation (TCFs) and self-concentration concept; it is assumed that the TCFs evolve on a much longer time scale than that of the segmental relaxation. This entails that the polymer blend can be viewed as a set of sub-volumes 'i' each with a different SBR concentration, $0 \leq \varphi_i \leq 1$. This quasi-static distribution of concentration $g(\varphi_i)$ in the blends can be described by a Gaussian function centered around the bulk concentration of the blend φ:

$$g(\varphi_i) \propto exp\frac{-(\varphi_i-\varphi)^2}{2\sigma^2} \tag{S4.5}$$

where σ is the standard deviation of the distribution of concentration.

Within each region we consider the effective concentration $\varphi_{eff,i}$ describing the fact that the dynamics of a given polymer segment in a blend is controlled by the local composition in a small region around that segment. This makes the concentration felt by each specific component to be higher than the average in this region. This effect is reflected by the corresponding self-concentration parameter, φ_{self}. Thus, the effective concentration $\varphi_{eff,i}$ in each region for the SBR and PS components is given by:

$$\varphi_{eff,i}^{SBR} = \varphi_{self}^{SBR} + \left(1 - \varphi_{self}^{SBR}\right)\varphi_i \tag{S4.6a}$$

$$\varphi_{eff,i}^{PS} = \varphi_{self}^{PS} + \left(1 - \varphi_{self}^{PS}\right)(1 - \varphi_i) \tag{S4.6b}$$

In this framework, the calorimetric behavior of SBR/PS blends is assumed to be the result of the superposition of contributions to the segmental heat capacity from different

regions, and within each region the result of the individual contributions from the blend components.[11] The contribution of each component in a region i of the blend is taken having the shape and amplitude corresponding to the pure component and weighted by its concentration. Thus, the contribution to the segmental heat capacity as a function of temperature for each component, can be calculated as:

$$s\text{-}C_p^{SBR}(T) = \sum g(\varphi_i)\, \varphi_i\, \Delta C_{pg}^{SBR} \left(\frac{T_{g,i}^{*,SBR}}{T}\right)^2 \frac{1}{1 + e^{(T_{g,i}^{*,SBR} - T)/\delta^{SBR}}} \qquad \text{(S4.7a)}$$

$$s\text{-}C_p^{PS}(T) = \sum g(\varphi_i)\, (1-\varphi_i)\, \Delta C_{pg}^{PS} \left(\frac{T_{g,i}^{*,PS}}{T}\right)^2 \frac{1}{1 + e^{(T_{g,i}^{*,PS} - T)/\delta^{PS}}} \qquad \text{(S4.7b)}$$

where we have assumed that in the description of the segmental heat capacity the only parameter affected by blending is $T_{g,i}^{*}$.

Therefore, the whole calorimetric signal can be obtained by summing up the respective contributions of SBR and PS:

$$s\text{-}C_{p,blend}(T) = s\text{-}C_p^{SBR}(T) + s\text{-}C_p^{PS}(T) \qquad \text{(S4.8)}$$

As a final step we will assume that the connections found for the homopolymer between the relaxation time and T_g^* remain valid in each region of the blend, i.e. $\tau_{SBR}\left(T_{g,i}^*\right) = \tau_g^{SBR}$ and $\tau_{PS}\left(T_{g,i}^*\right) = \tau_g^{PS}$. In this way $T_{g,i}^*$values appearing in equations S4.7a and S4.7b can be calculated using the VFT equation (eq. S4.1)[12-14] where the parameters D and T_0 correspond to those of region "i"

$$\tau_i(T) = \tau_\infty exp\left[D_i T_{0,i}/\left(T - T_{0,i}\right)\right]. \qquad \text{(S4.9)}$$

These parameters were obtained by using mixing rules with the corresponding effective concentrations. Particularly, a linear mixing rule is assumed for D_i:

$$D_i^{SBR} = D^{SBR}\varphi_{eff,i}^{SBR} + D^{PS}(1-\varphi_{eff,i}^{SBR}) \tag{S4.10a}$$

$$D_i^{PS} = D^{PS}\varphi_{eff,i}^{PS} + D^{SBR}(1-\varphi_{eff,i}^{PS}) \tag{S4.10b}$$

For $T_{0,i}$ we have used a Gordon-Taylor-like equation:

$$T_{0,i}^{SBR} = \frac{T_0^{PS}\left[\left(1-\varphi_{eff,i}^{SBR}\right)\right]+K_{G-T}\varphi_{eff,i}^{SBR}T_0^{SBR}}{\left(1-\varphi_{eff,i}^{SBR}\right)+K_{G-T}\varphi_{eff,i}^{SBR}} \tag{S4.11a}$$

$$T_{0,i}^{PS} = \frac{T_0^{PS}\varphi_{eff,i}^{PS}+K_{G-T}\left(1-\varphi_{eff,i}^{PS}\right)T_0^{SBR}}{\varphi_{eff,i}^{PS}+K_{G-T}\left(1-\varphi_{eff,i}^{PS}\right)} \tag{S4.11b}$$

After determining the $T_{g,i}^{*}$ values, the DSC curves can be described with the parameters above determined (Table S1). They were thus described in terms of those for the pure components and three fitting parameters: the self-concentrations of both components φ_{self}^{SBR} and φ_{self}^{PS} determining the local composition in each region, and the widths σ of the distributions of concentration associated to the spontaneous fluctuations, described by means of Gaussian functions, $g(\varphi_i)$. In previous work, the composition dependent values of σ and the values of φ_{self}^{SBR} and φ_{self}^{PS} were obtained from fitting the BDS experimental results, but in this work, we have analyzed the DSC curves of the three mixtures of each system allowing in a first step the three parameters, φ_{self}^{SBR} , φ_{self}^{PS} and σ, to vary freely. Figure S4.6 shows the steps needed to define the final parameter values: with the first step for each composition we have obtained the best values of φ_{self} for SBR and for PS. From these values we obtained the corresponding average self-concentration values $\varphi_{self}^{SBR} = 0.03$ and $\varphi_{self}^{PS} = 0.21$, which in the following will be taken composition independent. With these fixed values of φ_{self} we have run again the minimization routine

for the different compositions allowing only σ to change. We note that the relatively large uncertainties of the self-concentration in the analyzed systems are to a large extent related with the reduced dynamic asymmetry of these mixtures involving quite low molecular weight polystyrene oligomers. The smaller difference in the T_g values of the pure components reduces the experimental sensitivity to the self-concentration effects.

Finally, for each composition we have obtained the final σ values, composition dependent, σ=0.044 for the blend 80h, σ=0.108 for the blend 50h, σ=0.156 for the blend 20h, σ=0.065 for the blend 80d, σ=0.112 for the blend 50d, and σ=0.092 for the blend 20d. The resulting curves are shown in Figure S4.7 in comparison with the experimental data for the different blends investigated, where an overall excellent agreement between the two sets of data can be observed.

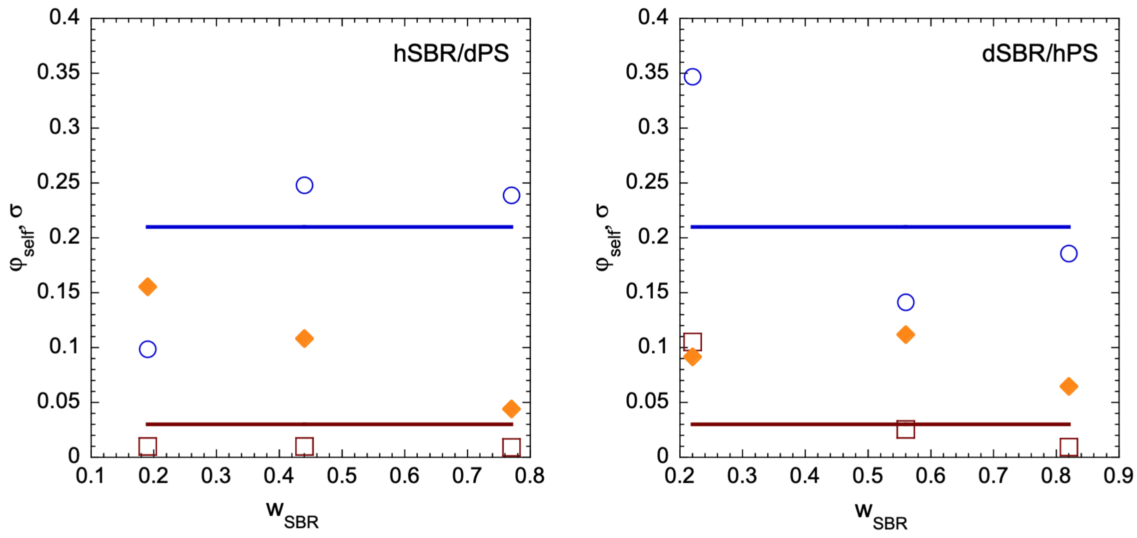

Figure S4.6. Evolution of the model parameter as function of the concentration of SBR in the blend. The red squares represent φ_{self}^{SBR} and the blue circles represent the φ_{self}^{PS} for each composition; solid lines in respective colors are the average value, $\varphi_{self}^{SBR} = 0.03$ and $\varphi_{self}^{PS} = 0.21$. Diamonds stand for σ values, composition dependent, for the different blends, keeping φ_{self}^{SBR} and φ_{self}^{PS} fixed.

Figure S4.8 shows the composition dependence of the width of the Gaussian distribution of concentration fluctuations deduced from the SANS results (described in the main manuscript) for four different values of the diameter of the explored sphere, $2R_c$, compared with the σ values obtained by the DSC modeling. From the comparison we can deduce that the relevant length scale for the α-relaxation would be of 30 ± 10 Å in these mixtures.

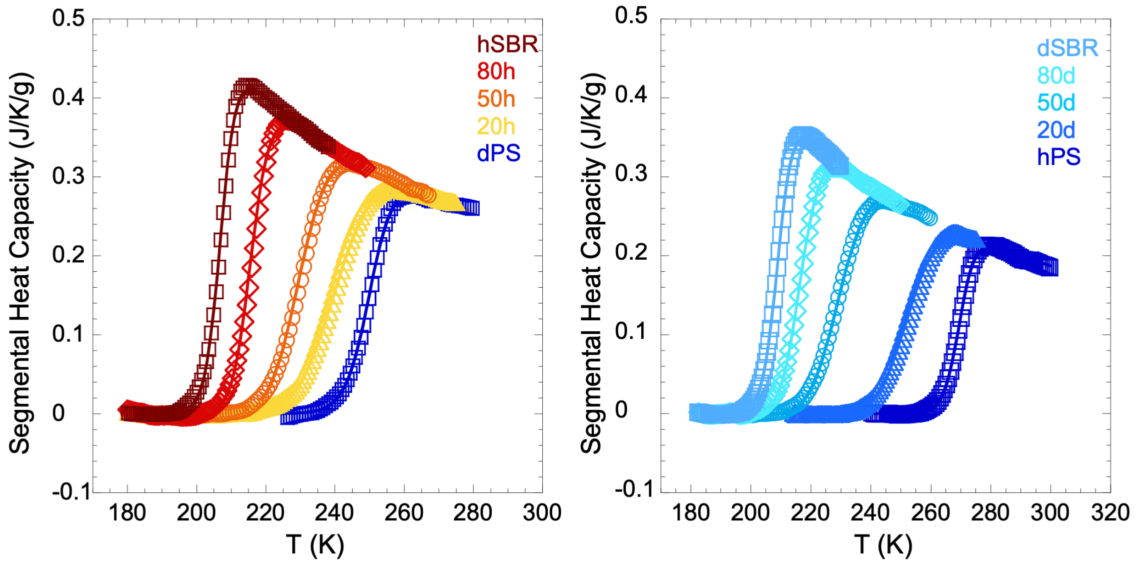

Figure S4.7. Segmental heat capacity for hSBR/dPS and dSBR/hPS blends. Solid lines stand for the output of the model.

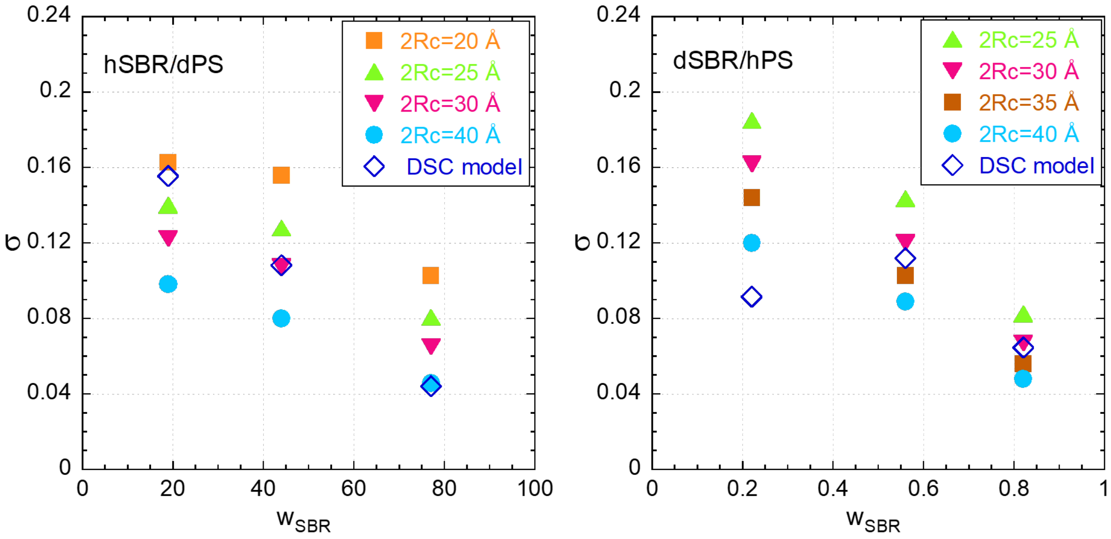

Figure S4.8. Concentration dependence of the width of the Gaussian distributions of concentration fluctuations deduced from the SANS results assuming different values for the relevant length scale $2R_c$ (filled symbols). Empty symbols correspond to values obtained by fitting DSC data (Fig. S4.7).

(vi) Evaluation of the components' effective T_g values

In Figure S4.9 the good agreement between the DSC trace and model is corroborated in the example for the $w_{hSBR} = 0.5$ blend, when the temperature derivative is compared. The good quality of the DSC data description is emphasized, both in peak position and in the breadth of the glass transition range. Figure S4.10 shows the direct comparison between experimental and calculated values of T_g as a function of blend composition, both series calculated from the inflection point of the segmental heat capacity *s*-$C_p(T)$ curves (peak temperatures in Figure S4.9). A very good agreement is obtained in this comparison. The whole set of data is very well described by the Gordon-Taylor equation (eq. S4.3).

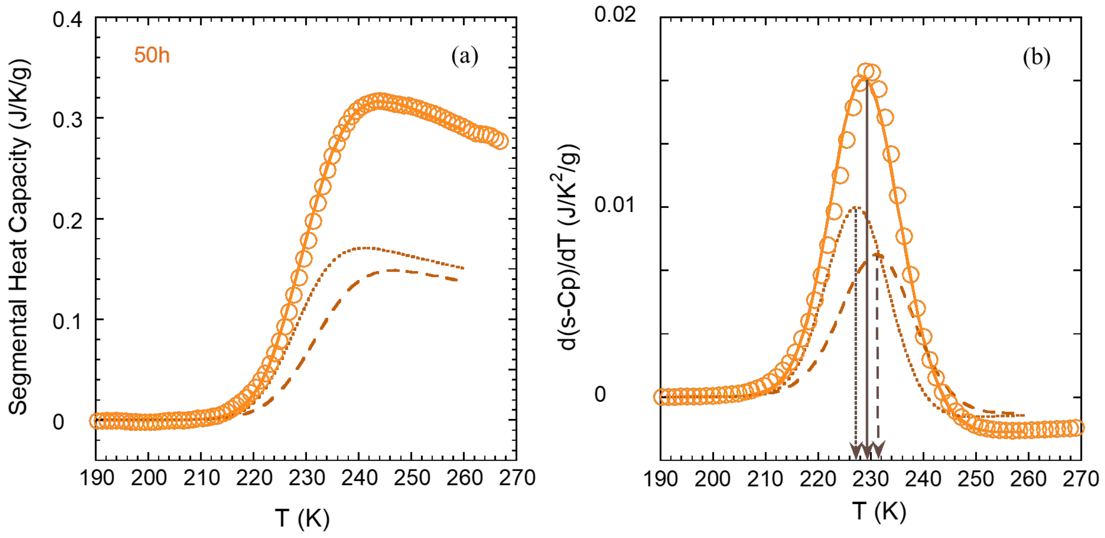

Figure S4.9. Segmental heat capacity (a) and temperature derivative of the segmental heat capacity (b) and corresponding output model (solid line) for the $w_{hSBR} = 0.5$; dashed lines show respectively the model contribution of SBR and PS components. Vertical arrows mark the corresponding values of T_g .

The modeling provides not only the overall DSC curves but also the individual contributions from SBR and PS components (see dashed and dotted lines in Figure S4.9). From the inflection point of the such calculated *s-C$_p$(T)* curves for the components, the so-called effective glass transition temperature[23] can be determined for each component of the blend. These effective $T_{g,eff}$ values have been included in Figure S4.10.

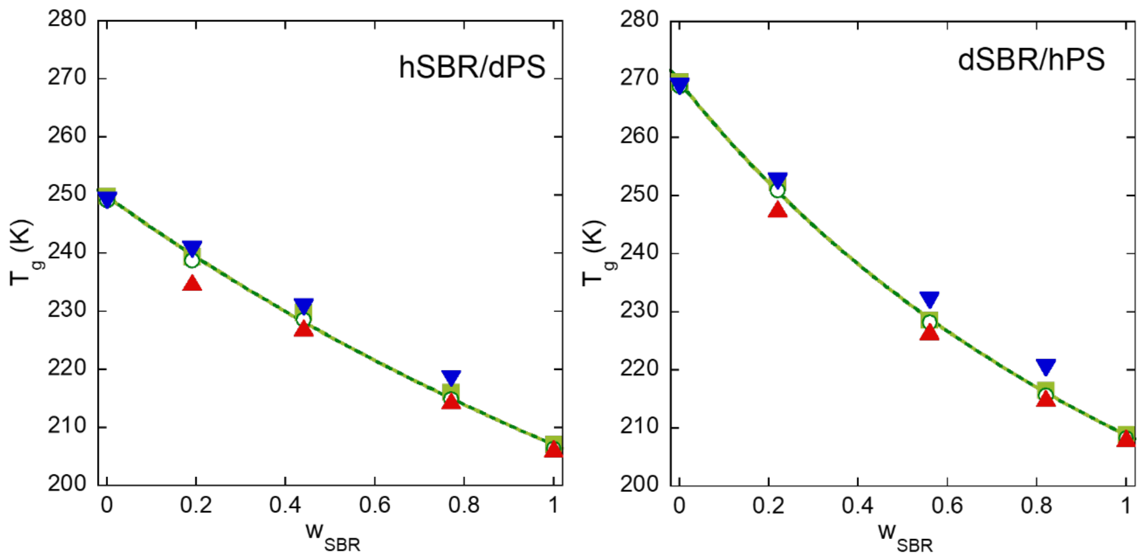

Figure S4.10. Comparison of the concentration dependence of the glass transition temperature as determined from the experimental curves (filled squares) and the whole model curve (empty circles). The lines are the prediction of the Gordon and Taylor equation (eq. S4.3) for the blends. The values of the effective glass-transitions of the components obtained from the model curves are shown as down-triangles for PS and up-triangles for SBR.

REFERENCES

(1) Schärpf, O. Polarization Analysis Techniques for Quasielastic Neutron Scattering. *Physica B: Condensed Matter* **1992**, *182*, 376-388.

(2) Zorn, R. On the evaluation of neutron scattering elastic scan data. *Nuclear Instruments and Methods in Physics Research Section A: Accelerators, Spectrometers, Detectors and Associated Equipment* **2009**, *603*, 439–445.

(3) Benedetto, A.; Kearley, G. Experimental demonstration of the novel "van-Hove integral method (vHI)" for measuring diffusive dynamics by elastic neutron scattering. *Scientific Reports* **2021**, *11*, 14093.

(4) Doster, W.; Diehl, M.; Petry, W.; Ferrand, M. Elastic resolution spectroscopy: a method to study molecular motions in small biological samples. *Physica B: Condensed Matter* **2001**, *301*, 65–68.

(5) Ayyagari, C.; Bedrov, D.; Smith, G. D. Structure of atactic polystyrene: a Molecular Dynamics simulation study. *Macromolecules* **2000**, *33*, 6194-6199.

(6) Moreno, A. J.; Arbe, A.; Colmenero, J. Structure and dynamics of self-assembled comb copolymers: comparison between simulations of a generic model and neutron scattering experiments. *Macromolecules* **2011**, *44*, 1695-1706.

(7) Bhowmick, D.; Pomposo, J.A.; Juranyi, F.; García-Sakai, V.; Zamponi, M.; Su, Y.; Arbe, A.; Colmenero, J. Microscopic dynamics in nanocomposites of poly(ethylene oxide) and poly(methyl methacrylate) soft nanoparticles: a quasi-elastic neutron scattering study. *Macromolecules* **2014**, *47*, 304-315.

(8) Bhowmick, D.; Pomposo, J.A.; Juranyi, F.; García-Sakai, V.; Zamponi, M.; Su, Y.; Arbe, A.; Colmenero, J. Investigation of a nanocomposite of 75 wt % poly(methyl methacrylate) nanoparticles with 25 wt % poly(ethylene oxide) linear chains: a quasielatic neutron scattering, calorimetric, and WAXS study. *Macromolecules* **2014**, *47*, 3005-3016.

(9) Alvarez, F.; Alegría, A.; Colmenero, J. Interconnection between Frequency-Domain Havriliak-Negami and Time-Domain Kohlrausch-Williams-Watts Relaxation Functions. *Physical Review B* **1993**, *47*, 125–130.

(10) Kremer, A., Friedrich; Schönhals, Ed. Broadband Dielectric Spectroscopy; Springer: **2003**.

(11) Shafqat, N.; Alegría, A.; Arbe, A.; Malicki, N.; Dronet, S.; Porcar L.; Colmenero J. Disentangling the calorimetric glass-transition trace in polymer/oligomer mixtures from the modeling of dielectric relaxation and the input of small-angle neutron scattering. *Macromolecules* **2022**, *55*, 7614-7625.

(12) Vogel, H. The Law of the Relation between the Viscosity of Liquids and the Temperature. *Physikalische Zeitschrift* **1921**, *22*, 645.

(13) Fulcher, G. S. Analysis of Recent Measurements of the Viscosity of Glasses. Journal of the American Ceramic Society **1925**, *8*, 339–355.

(14) Tammann, G.; Hesse, W. Die Abhängigkeit der Viscosität von der Temperatur bei unterkühlten Flüssigkeiten. *Zeitschrift für anorganische und allgemeine Chemie* **1926**, *156*, 245–257.

(15) Lindon, W.; Ranganathan, V.; Douglas, R. MacF.; Gloria, D. E. Application of the Kwei equation to model the T_g behavior of binary blends of sugars and salts. *Cryobiology* **2014**, *68*, 155-158.

(16) Gordon, M.; Taylor, JS. Ideal copolymers and the second - order transitions of synthetic rubbers. i. non - crystalline copolymers. *J Appl Chem*. **1952**, *2*, 493–500.

(17) Brostow, W.; Chiu, R.; Kalogeras, IM.; Vassilikou-Dova, A. Prediction of glass transition temperatures: binary blends and copolymers. *Mater Lett.* **2008**, *62*, 3152–3155.

(18) Fox, T.G. Influence of Diluent and of Copolymer Composition on the Glass Temperature of a Polymer System. *Bull. Am. Phys. Soc*. **1956**, 7280-7288.

(19) Couchman, P.; Karasz, F. A classical thermodynamic discussion of the effect of composition on glass transition temperatures. *Macromolecules* **1978**, *11*, 117–119.

(20) Katkov, I. I.; and Levine, F. Prediction of the glass transition temperature of water solutions: comparison of different models. *Cryobiology* **2004**, *49*, 62–82 (2004).

(21) Gambino, T.; Alegría, A.; Arbe, A.; Colmenero, J.; Malicki, N.; Dronet, S.; Schnell, B.; Lohstroh, W.; Nemkovski, K. Applying polymer blend dynamics concepts to a simplified industrial system. A combined effort by dielectric spectroscopy and neutron scattering. *Macromolecules* **2018**, *51*, 6692–6706.

(22) Gambino, T.; Alegría, A.; Arbe, A.; Colmenero, J.; Malicki, N.; Dronet, S. Modeling the high frequency mechanical relaxation of simplified industrial polymer mixtures using dielectric relaxation results. *Polymer* **2020**, 187, 122051.

(23) Lodge, T. P.; McLeish, T. C. B. Self-concentration and effective glass transition temperature in polymer blends. *Macromolecules* **2000**, 33, 5278-5284.
